# Supplementary material for: Transcriptomic profiling of Debaryomyces hansenii reveals detoxification and stress responses to benzo(a)pyrene exposure
Source: Appl Environ Microbiol. 2025 Sep 16;91(10):e01557-25. doi: 10.1128/aem.01557-25 (PMC12542653; doi:10.1128/aem.01557-25)
Supplement: Figure S1 — Growth curves, dry weight, BaP degradation, and glucose consumption of Debaryomyces hansenii cultures under the indicated conditions. [file aem.01557-25-s0001.pdf]

Growth curves

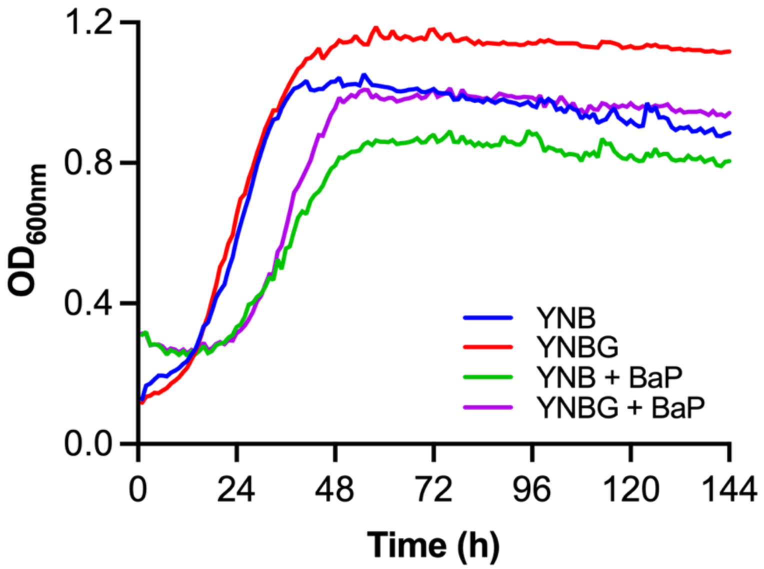

Data are shown as mean ± SD from three biological replicates, each calculated from 10 technical measurements (n = 30). Areas under the curve (AUC) were calculated for each biological replicate and used for statistical analysis. One-way ANOVA was performed assuming normal distribution and equal variances, followed by Tukey's multiple comparisons test. Differences were considered statistically significant at $p < 0.05$. Adjusted p-values: ns ($p = 0.1234$), **** ($p < 0.0001$).

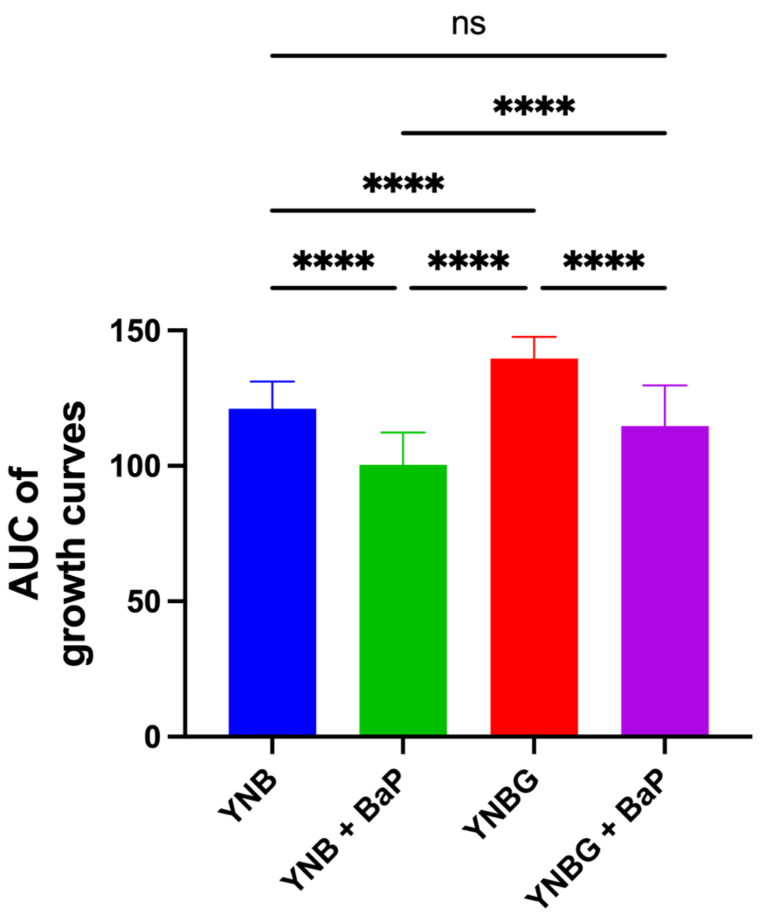

Dry weight

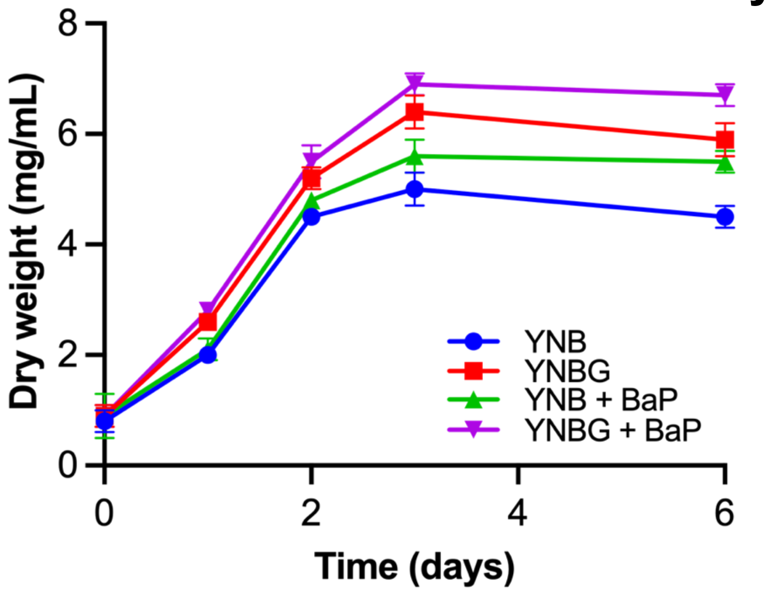

Data are shown as mean ± SD from three biological replicates, each calculated from 6 technical measurements (n = 18). Areas under the curve (AUC) were calculated for each biological replicate and used for statistical analysis. One-way ANOVA was performed assuming normal distribution and equal variances, followed by Tukey's multiple comparisons test. Differences were considered statistically significant at $p < 0.05$. Adjusted p-values: ns ($p = 0.1234$), **** ($p < 0.0001$).

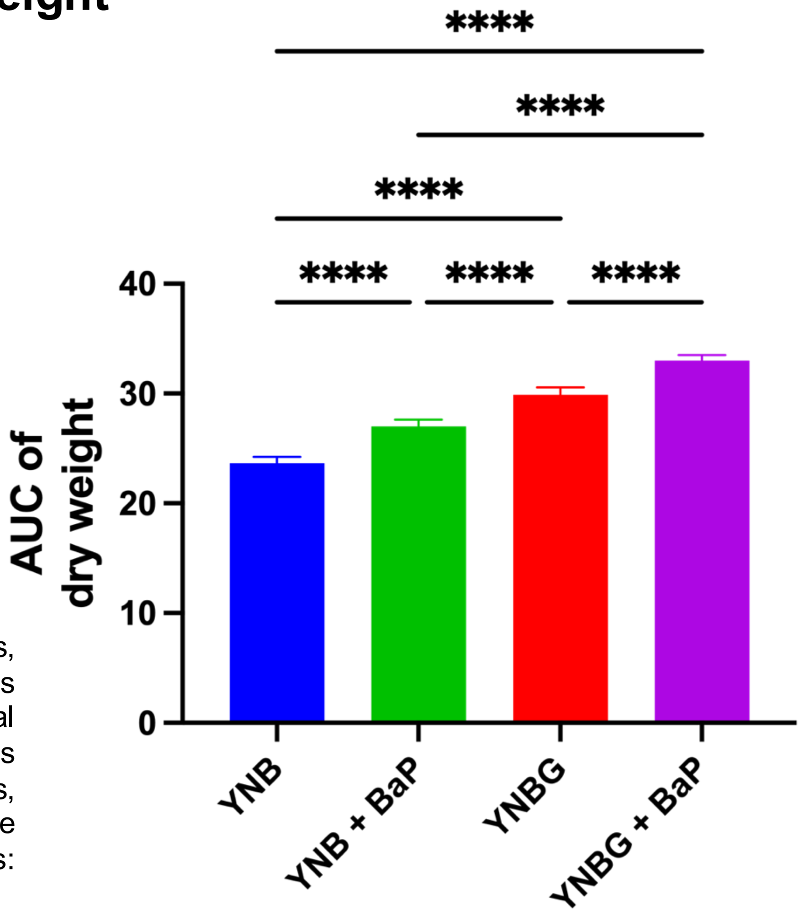

BaP degradation

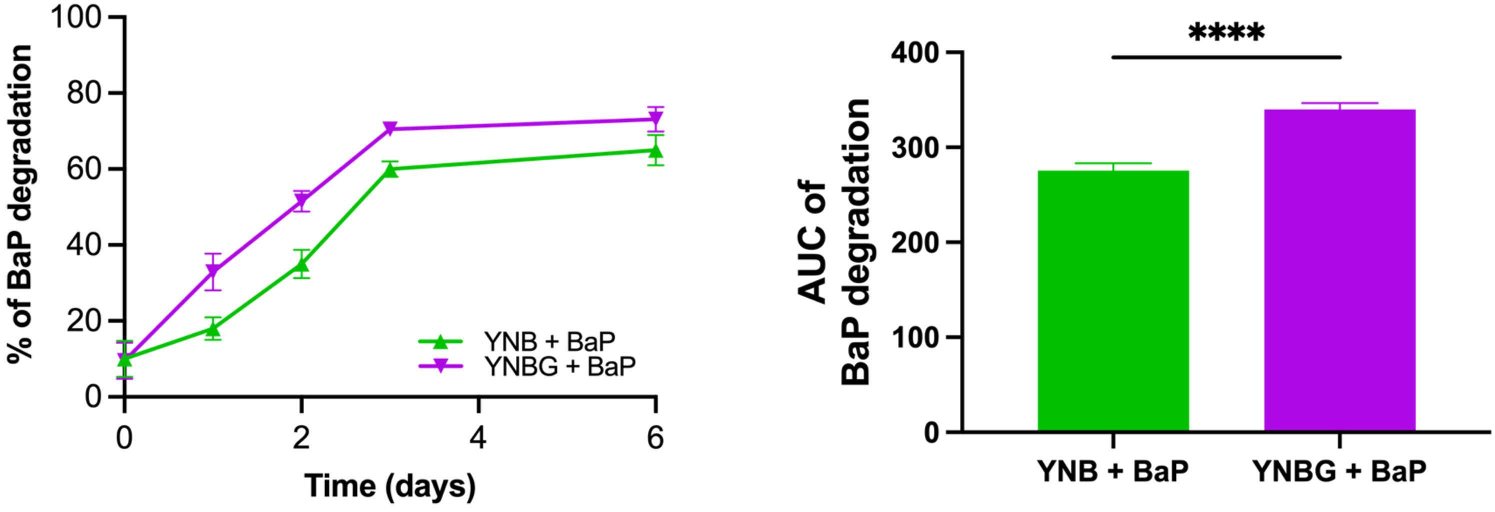

Data are presented as mean ± SD from three biological replicates, each derived from 3 technical measurements (n = 9). AUC values were calculated for each biological replicate and analyzed using an unpaired Student’s t-test, assuming normal distribution and equal variances. Statistical significance was set at $p < 0.05$. Adjusted p-value: **** ($p < 0.0001$).

Glucose consumption

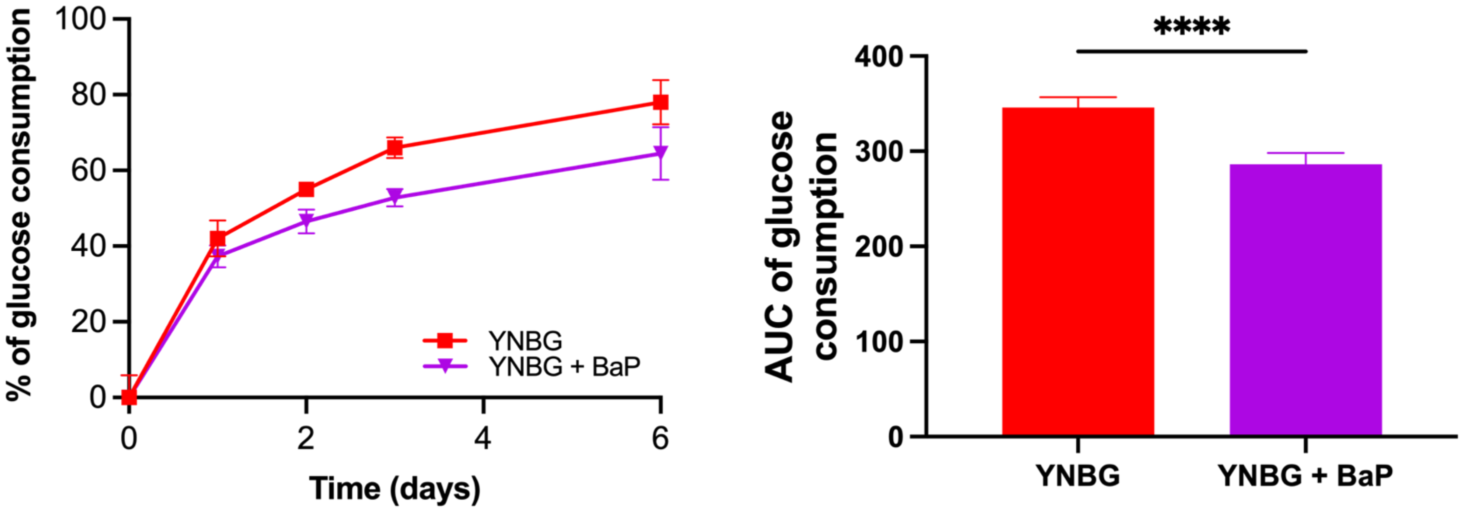

Data are presented as mean ± SD from three biological replicates, each based on 3 technical measurements (n = 9). AUC values were computed per biological replicate and analyzed using an unpaired Student’s t-test under the assumption of normality and equal variances. Differences were considered significant at $p < 0.05$. Adjusted p-value: **** ($p < 0.0001$).

Supplementary Figure 1. Growth curves, dry weight, BaP degradation, and glucose consumption of *Debaryomyces hansenii* cultures under the indicated conditions. Data are shown as mean ± SD from three biological replicates, each derived from multiple technical measurements ($n = 9–30$, depending on the assay). Areas under the curve (AUC) were calculated per biological replicate and used for statistical analysis. Growth curves and dry weight were analyzed by one-way ANOVA with Tukey's post hoc test, while BaP degradation and glucose consumption were analyzed by unpaired Student's t-test. Differences were considered statistically significant at $p < 0.05$. Adjusted p-values: ns ($p = 0.1234$), **** ($p < 0.0001$).
