# Supplementary material for: Transcriptomic profiling of Debaryomyces hansenii reveals detoxification and stress responses to benzo(a)pyrene exposure
Source: Appl Environ Microbiol. 2025 Sep 16;91(10):e01557-25. doi: 10.1128/aem.01557-25 (PMC12542653; doi:10.1128/aem.01557-25)
Supplement: Figure S2 — Representative GC-MS chromatograms of BaP controls and degradation assays. [file aem.01557-25-s0002.pdf]

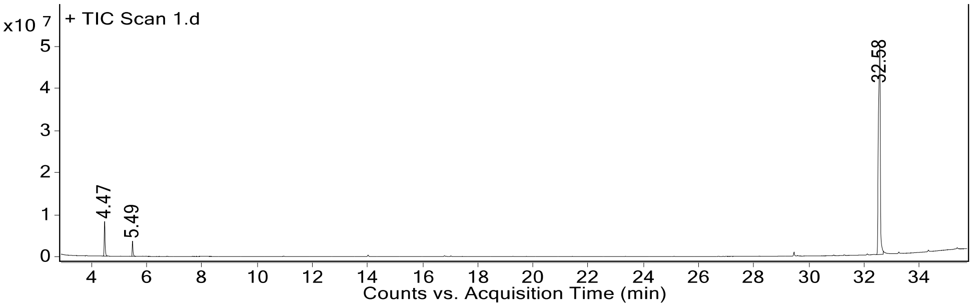

a) 100 ppm of BaP dissolved in acetone

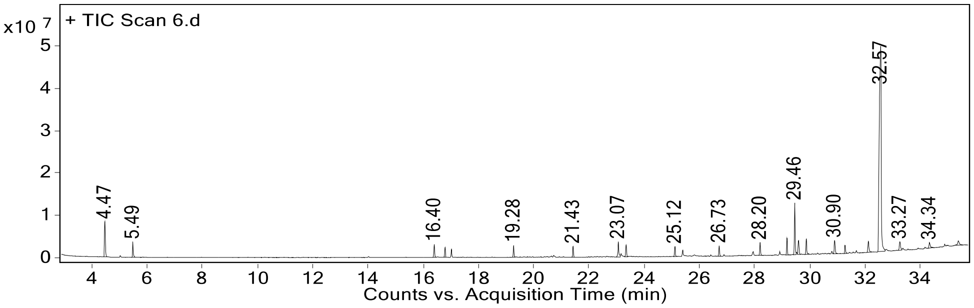

b) 100 ppm of BaP extracted from YNB

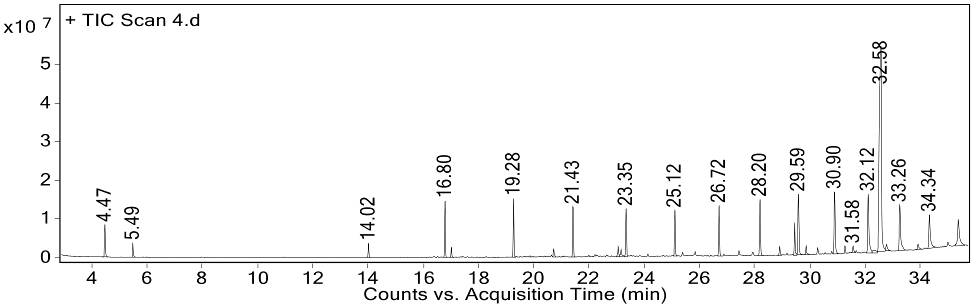

c) 100 ppm of BaP extracted from YNB medium with dead inoculum

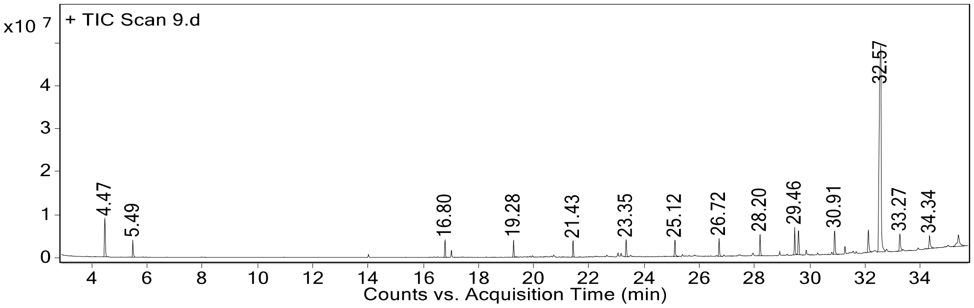

d) Day 6 degradation in YNB medium with 100 ppm of BaP

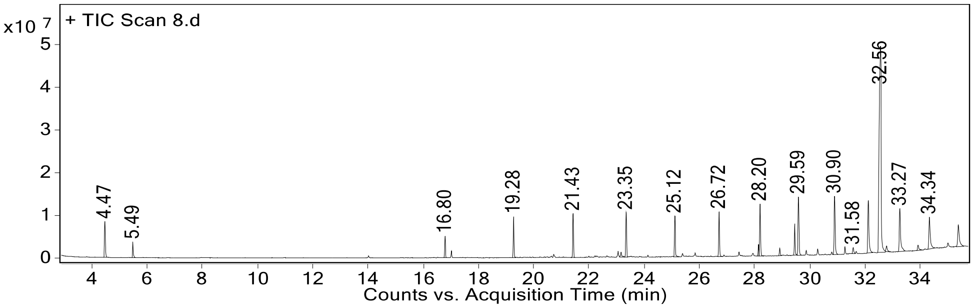

e) 100 ppm of BaP extracted from YNBG medium with dead inoculum

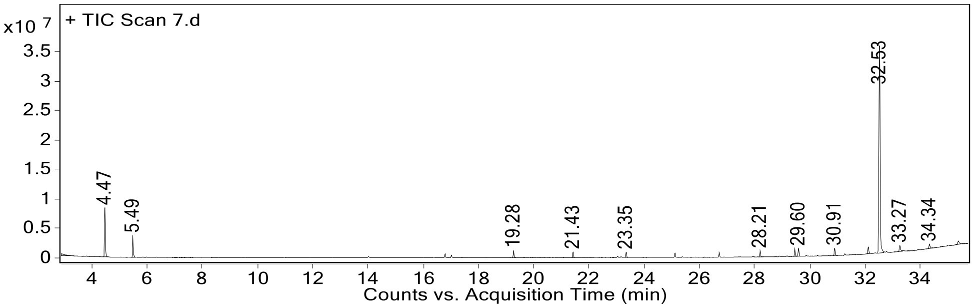

f) Day 6 degradation in YNBG medium with 100 ppm of BaP

Supplementary Figure 2. Representative GC–MS chromatograms of BaP controls and degradation assays. (a) Standard of 100 ppm BaP dissolved in acetone. (b) Extraction of 100 ppm BaP from YNB medium. (c) Extraction of 100 ppm BaP from YNB medium with dead inoculum. (d) BaP degradation in YNB medium with 100 ppm BaP after 6 days. (e) Extraction of 100 ppm BaP from YNBG medium with dead inoculum. (f) BaP degradation in YNBG medium with 100 ppm BaP after 6 days.
