# Supplementary material for: Transcriptomic profiling of Debaryomyces hansenii reveals detoxification and stress responses to benzo(a)pyrene exposure
Source: Appl Environ Microbiol. 2025 Sep 16;91(10):e01557-25. doi: 10.1128/aem.01557-25 (PMC12542653; doi:10.1128/aem.01557-25)
Supplement: Figure S3 — Predicted pathways associated with BaP degradation (YNB + BaP) and stress response in Debaryomyces hansenii. [file aem.01557-25-s0003.pdf]

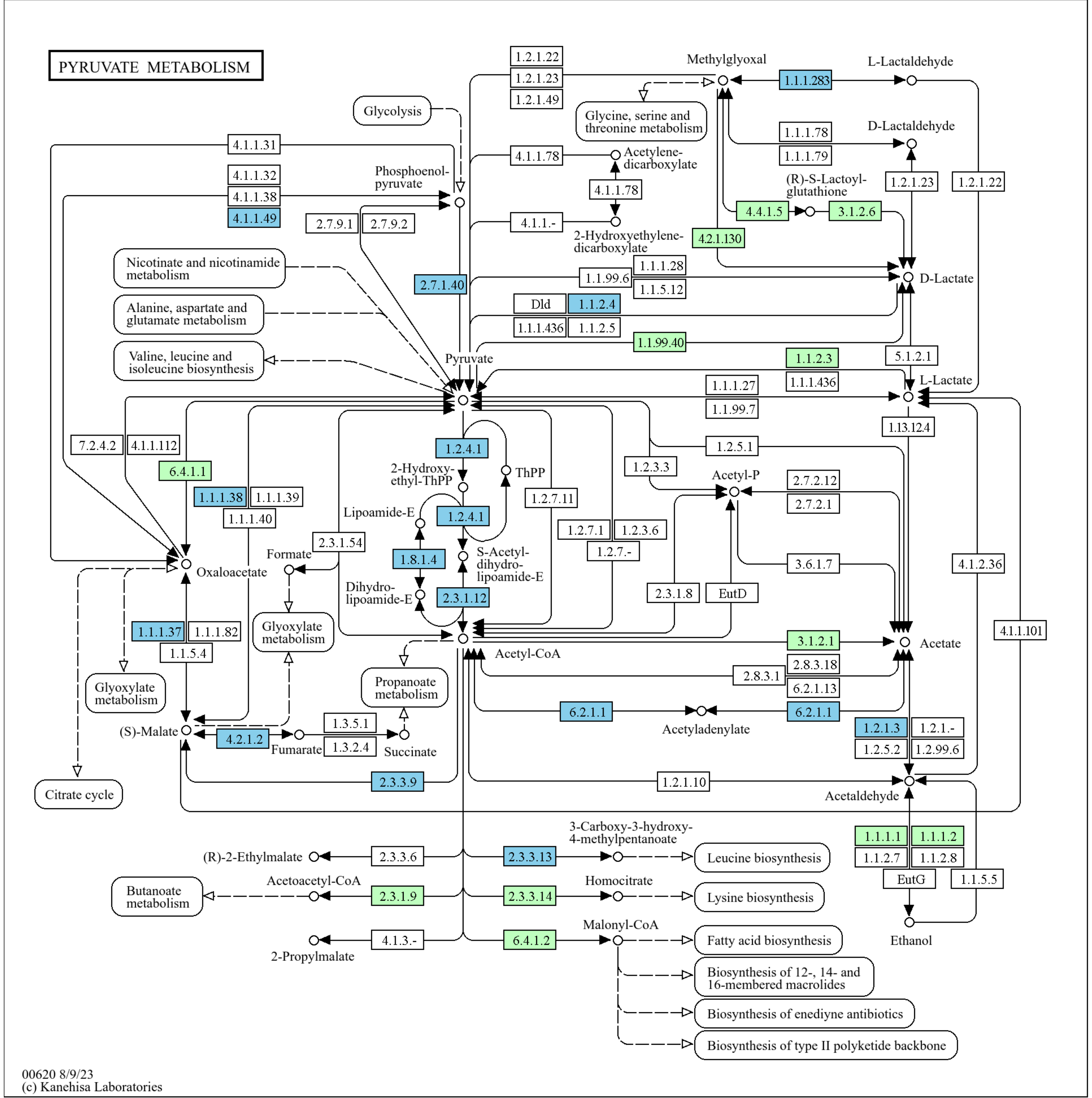


In all figures, white indicates enzymes absent in *Debaryomyces hansenii*, green indicates those present but not expressed, and blue indicates those that were overexpressed.

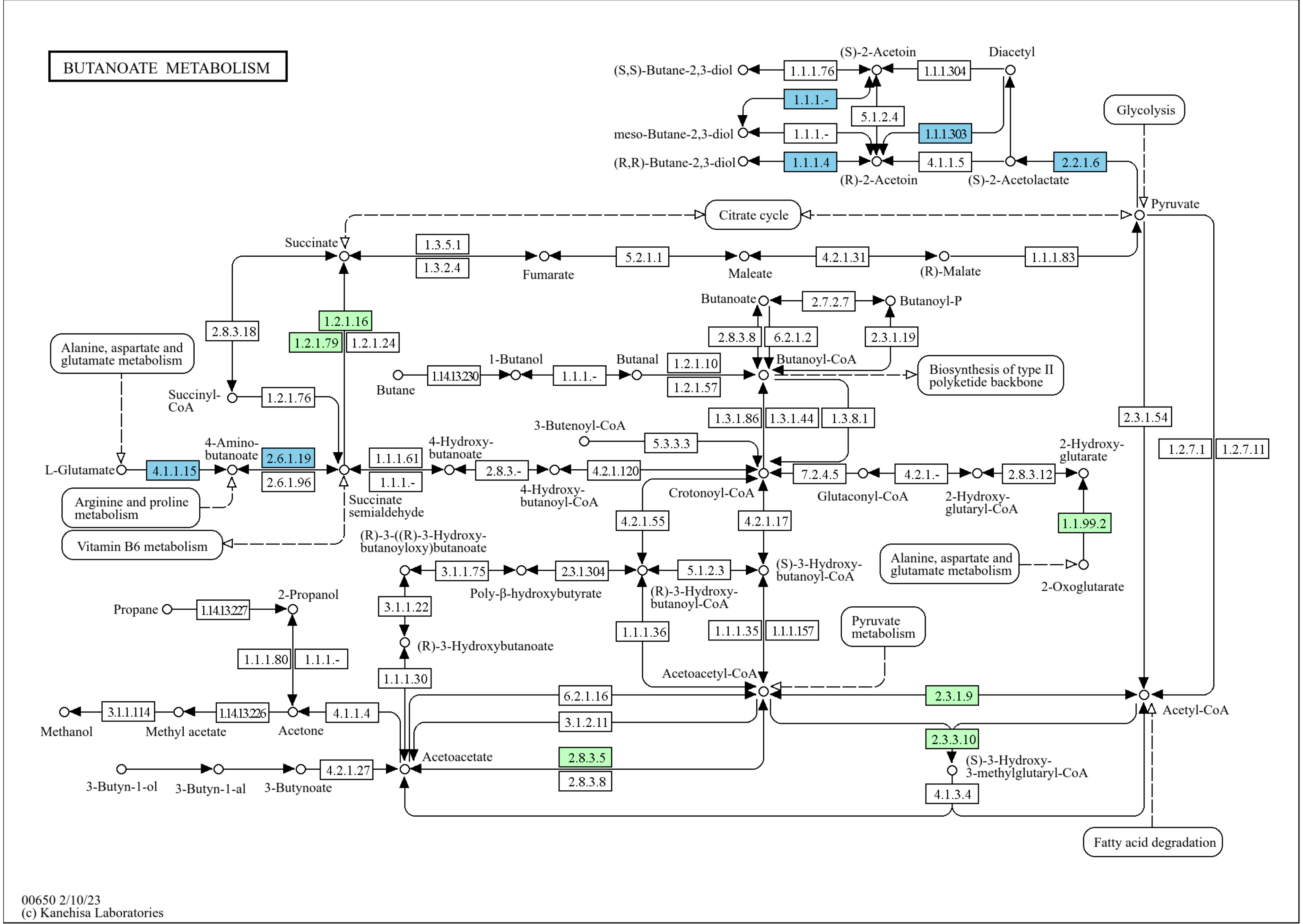
BUTANOATE METABOLISM
(S,S)-Butane-2,3-diol
1.1.1.76
(S)-2-Acetoin
1.1.1.304
Diacetyl
1.1.1.-
5.1.2.4
meso-Butane-2,3-diol
1.1.1.-
1.1.1.303
(R,R)-Butane-2,3-diol
1.1.1.4
4.1.1.5
2.2.1.6
(R)-2-Acetoin
(S)-2-Acetolactate
Glycolysis
Pyruvate
Citrate cycle
Succinate
1.3.5.1
1.3.2.4
Fumarate
5.2.1.1
Maleate
4.2.1.31
(R)-Malate
1.1.1.83
Butanoate
2.7.2.7
Butanoyl-P
2.8.3.18
1.2.1.16
1.2.1.79
1.2.1.24
2.8.3.8
6.2.1.2
2.3.1.19
Alanine, aspartate and glutamate metabolism
1-Butanol
Butanal
1.2.1.10
Butanoyl-CoA
Biosynthesis of type II polyketide backbone
Butane
1.14.13.230
1.1.1.-
1.2.1.57
Succinyl-CoA
1.2.1.76
1.3.1.86
1.3.1.44
1.3.8.1
2.3.1.54
3-Butenoyl-CoA
5.3.3.3
4-Amino-butanoate
2.6.1.19
4-Hydroxy-butanoate
1.1.1.61
2-Hydroxy-glutarate
1.2.7.1
1.2.7.11
L-Glutamate
4.1.1.15
2.6.1.96
1.1.1.-
2.8.3.-
4.2.1.120
7.2.4.5
4.2.1.-
2.8.3.12
Arginine and proline metabolism
Succinate semialdehyde
4-Hydroxy-butanoyl-CoA
Crotonoyl-CoA
Glutaconyl-CoA
2-Hydroxy-glutaryl-CoA
Vitamin B6 metabolism
4.2.1.55
4.2.1.17
1.1.99.2
(R)-3-((R)-3-Hydroxy-butanoyloxy)butanoate
3.1.1.75
Poly-β-hydroxybutyrate
2.3.1.304
5.1.2.3
(S)-3-Hydroxy-butanoyl-CoA
Alanine, aspartate and glutamate metabolism
2-Oxoglutarate
(R)-3-Hydroxy-butanoyl-CoA
2-Propanol
Propane
1.14.13.227
3.1.1.22
(R)-3-Hydroxybutanoate
1.1.1.36
1.1.1.35
1.1.1.157
Pyruvate metabolism
1.1.1.80
1.1.1.-
1.1.1.30
Acetoacetyl-CoA
6.2.1.16
2.3.1.9
Methanol
3.1.1.114
Methyl acetate
1.14.13.226
Acetone
4.1.1.4
3.1.2.11
Acetyl-CoA
2.3.3.10
Acetoacetate
2.8.3.5
(S)-3-Hydroxy-3-methylglutaryl-CoA
3-Butyn-1-ol
3-Butyn-1-al
3-Butynoate
4.2.1.27
2.8.3.8
4.1.3.4
Fatty acid degradation
00650 2/10/23
(c) Kanehisa Laboratories

# AMINO SUGAR AND NUCLEOTIDE SUGAR METABOLISM

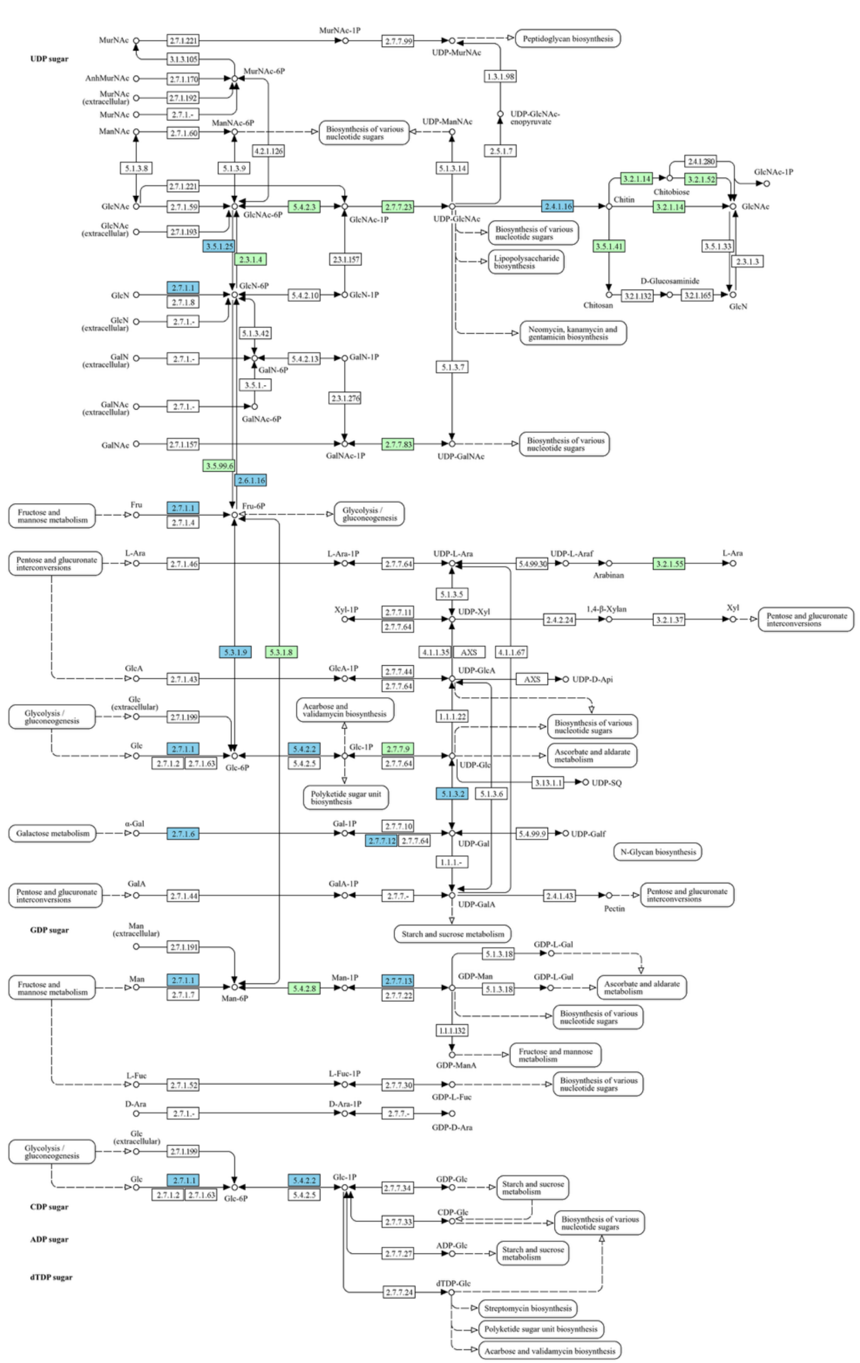


00520 4/4/25
(c) Kanehisa Laboratories

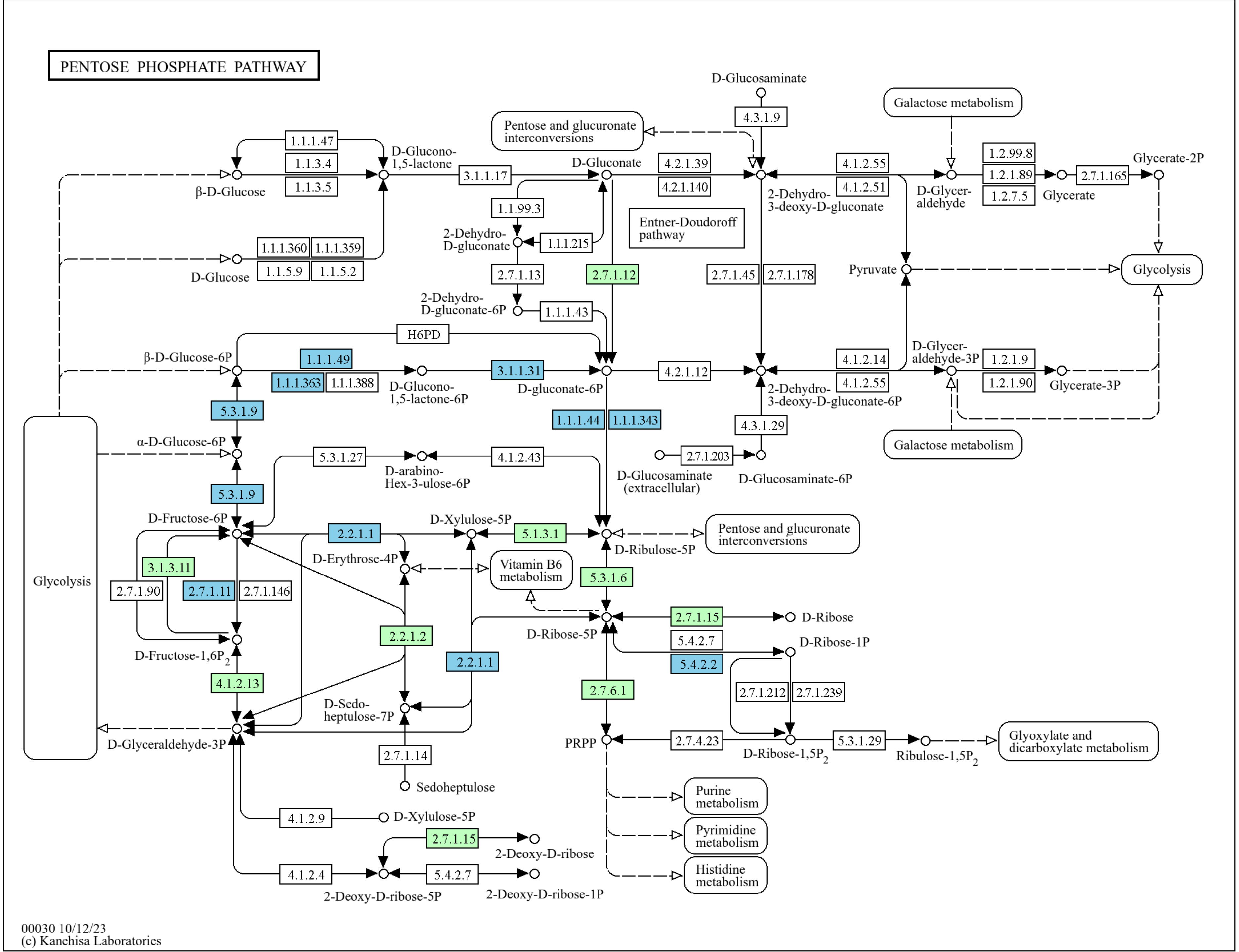
PENTOSE PHOSPHATE PATHWAY
D-Glucosaminate
4.3.1.9
Galactose metabolism
Pentose and glucuronate interconversions
1.1.1.47
1.1.3.4
1.1.3.5
D-Glucono-1,5-lactone
3.1.1.17
D-Gluconate
4.2.1.39
4.2.1.140
4.1.2.55
4.1.2.51
1.2.99.8
1.2.1.89
1.2.7.5
2.7.1.165
Glycerate-2P
β-D-Glucose
2-Dehydro-3-deoxy-D-gluconate
D-Glyceraldehyde
Glycerate
1.1.99.3
Entner-Doudoroff pathway
2-Dehydro-D-gluconate
1.1.1.215
1.1.1.360
1.1.1.359
1.1.5.9
1.1.5.2
D-Glucose
2.7.1.13
2.7.1.12
2.7.1.45
2.7.1.178
Pyruvate
Glycolysis
2-Dehydro-D-gluconate-6P
1.1.1.43
H6PD
D-Glyceraldehyde-3P
β-D-Glucose-6P
1.1.1.49
3.1.1.31
4.2.1.12
4.1.2.14
4.1.2.55
1.2.1.9
1.2.1.90
Glycerate-3P
1.1.1.363
1.1.1.388
D-Glucono-1,5-lactone-6P
D-gluconate-6P
2-Dehydro-3-deoxy-D-gluconate-6P
5.3.1.9
1.1.1.44
1.1.1.343
4.3.1.29
Galactose metabolism
α-D-Glucose-6P
5.3.1.27
4.1.2.43
2.7.1.203
D-arabino-Hex-3-ulose-6P
D-Glucosaminate (extracellular)
D-Glucosaminate-6P
5.3.1.9
D-Fructose-6P
D-Xylulose-5P
2.2.1.1
5.1.3.1
D-Ribulose-5P
Pentose and glucuronate interconversions
D-Erythrose-4P
Vitamin B6 metabolism
3.1.3.11
5.3.1.6
Glycolysis
2.7.1.90
2.7.1.11
2.7.1.146
2.7.1.15
D-Ribose
D-Ribose-5P
5.4.2.7
D-Ribose-1P
2.2.1.2
5.4.2.2
D-Fructose-1,6P2
2.2.1.1
4.1.2.13
2.7.6.1
2.7.1.212
2.7.1.239
D-Sedo-heptulose-7P
PRPP
2.7.4.23
5.3.1.29
Glyoxylate and dicarboxylate metabolism
D-Glyceraldehyde-3P
D-Ribose-1,5P2
Ribulose-1,5P2
2.7.1.14
Sedoheptulose
Purine metabolism
4.1.2.9
D-Xylulose-5P
Pyrimidine metabolism
2.7.1.15
2-Deoxy-D-ribose
4.1.2.4
5.4.2.7
Histidine metabolism
2-Deoxy-D-ribose-5P
2-Deoxy-D-ribose-1P
00030 10/12/23
(c) Kanehisa Laboratories

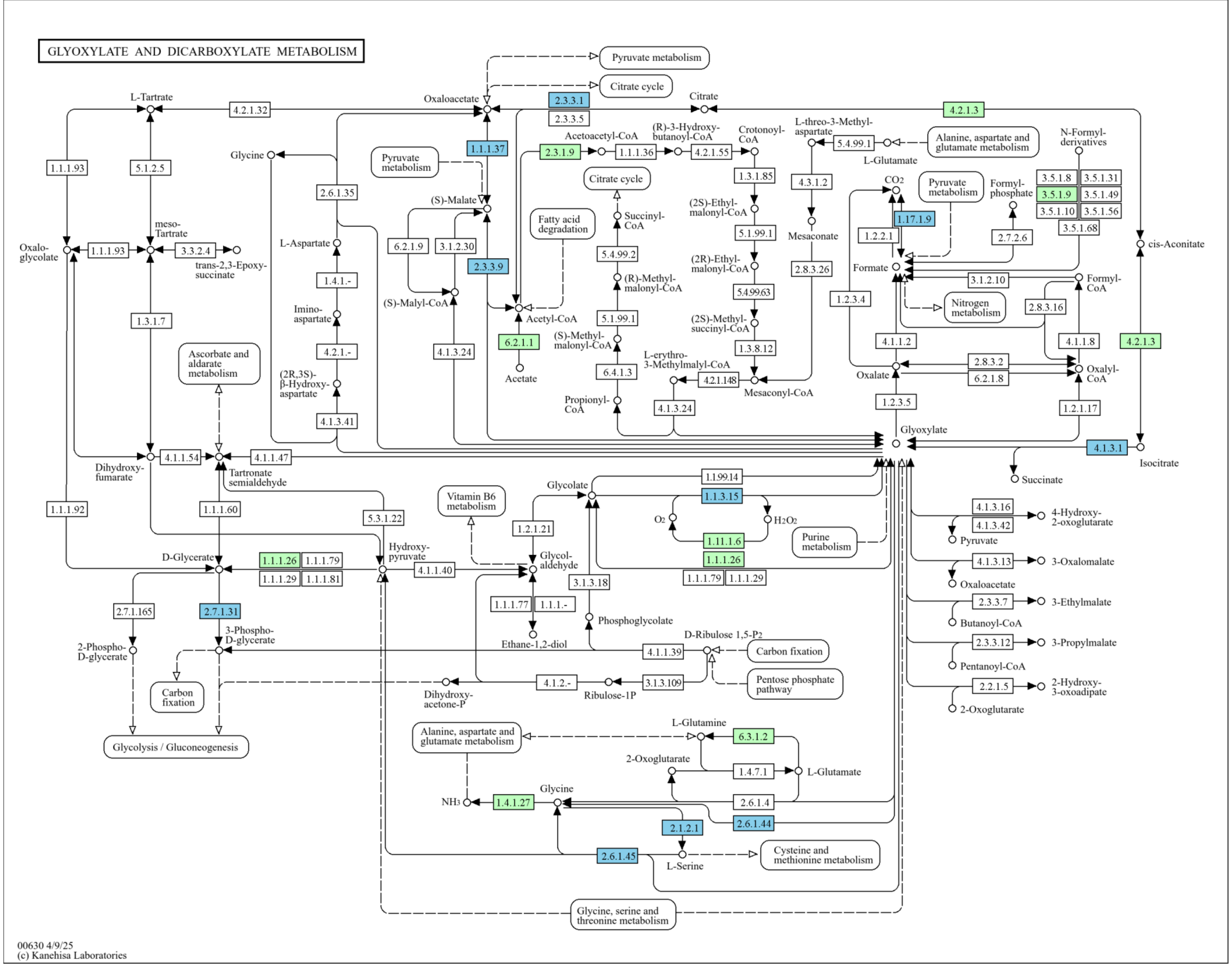
GLYOXYLATE AND DICARBOXYLATE METABOLISM
Pyruvate metabolism
Citrate cycle
L-Tartrate
4.2.1.32
Oxaloacetate
2.3.3.1
2.3.3.5
Citrate
4.2.1.3
Glycine
Acetoacetyl-CoA
(R)-3-Hydroxy-butanoyl-CoA
Crotonoyl-CoA
L-threo-3-Methyl-aspartate
Alanine, aspartate and glutamate metabolism
N-Formyl-derivatives
1.1.1.93
5.1.2.5
2.6.1.35
Pyruvate metabolism
1.1.1.37
2.3.1.9
1.1.1.36
4.2.1.55
5.4.99.1
L-Glutamate
Citrate cycle
1.3.1.85
4.3.1.2
CO2
Pyruvate metabolism
Formyl-phosphate
3.5.1.8
3.5.1.31
3.5.1.9
3.5.1.49
3.5.1.10
3.5.1.56
3.5.1.68
(S)-Malate
Fatty acid degradation
Succinyl-CoA
(2S)-Ethyl-malonyl-CoA
Mesaconate
1.17.1.9
2.7.2.6
meso-Tartrate
L-Aspartate
6.2.1.9
3.1.2.30
5.1.99.1
1.2.2.1
Oxalo-glycolate
1.1.1.93
3.3.2.4
trans-2,3-Epoxy-succinate
2.3.3.9
5.4.99.2
(2R)-Ethyl-malonyl-CoA
2.8.3.26
Formate
cis-Aconitate
1.4.1.-
(R)-Methyl-malonyl-CoA
3.1.2.10
Formyl-CoA
(S)-Malyl-CoA
5.4.99.63
1.2.3.4
Nitrogen metabolism
2.8.3.16
Imino-aspartate
Acetyl-CoA
5.1.99.1
(2S)-Methyl-succinyl-CoA
1.3.1.7
Ascorbate and aldarate metabolism
6.2.1.1
(S)-Methyl-malonyl-CoA
4.1.1.2
4.1.1.8
4.2.1.3
4.2.1.-
4.1.3.24
1.3.8.12
L-erythro-3-Methylmalyl-CoA
2.8.3.2
Oxalyl-CoA
(2R,3S)-β-Hydroxy-aspartate
Acetate
6.4.1.3
4.2.1.148
Mesaconyl-CoA
Oxalate
6.2.1.8
Propionyl-CoA
4.1.3.41
4.1.3.24
1.2.3.5
1.2.1.17
Glyoxylate
4.1.1.54
4.1.1.47
4.1.3.1
Isocitrate
Dihydroxy-fumarate
Tartronate semialdehyde
Succinate
1.1.99.14
Glycolate
Vitamin B6 metabolism
1.1.3.15
1.1.1.92
1.1.1.60
5.3.1.22
O2
H2O2
4.1.3.16
4-Hydroxy-2-oxoglutarate
1.2.1.21
4.1.3.42
Pyruvate
Purine metabolism
1.11.1.6
Hydroxy-pyruvate
Glycol-aldehyde
D-Glycerate
1.1.1.26
1.1.1.79
4.1.1.40
1.1.1.26
4.1.3.13
3-Oxalomalate
1.1.1.29
1.1.1.81
3.1.3.18
1.1.1.79
1.1.1.29
Oxaloacetate
2.7.1.165
2.7.1.31
1.1.1.77
1.1.1.-
2.3.3.7
3-Ethylmalate
Phosphoglycolate
Butanoyl-CoA
3-Phospho-D-glycerate
Ethane-1,2-diol
D-Ribulose 1,5-P2
2-Phospho-D-glycerate
4.1.1.39
Carbon fixation
2.3.3.12
3-Propylmalate
Pentanoyl-CoA
Carbon fixation
4.1.2.-
3.1.3.109
Pentose phosphate pathway
2.2.1.5
2-Hydroxy-3-oxoadipate
Dihydroxy-acetone-P
Ribulose-1P
2-Oxoglutarate
Glycolysis / Gluconeogenesis
Alanine, aspartate and glutamate metabolism
L-Glutamine
6.3.1.2
2-Oxoglutarate
1.4.7.1
L-Glutamate
Glycine
NH3
1.4.1.27
2.6.1.4
2.6.1.44
2.1.2.1
2.6.1.45
Cysteine and methionine metabolism
L-Serine
Glycine, serine and threonine metabolism
00630 4/9/25
(c) Kanehisa Laboratories

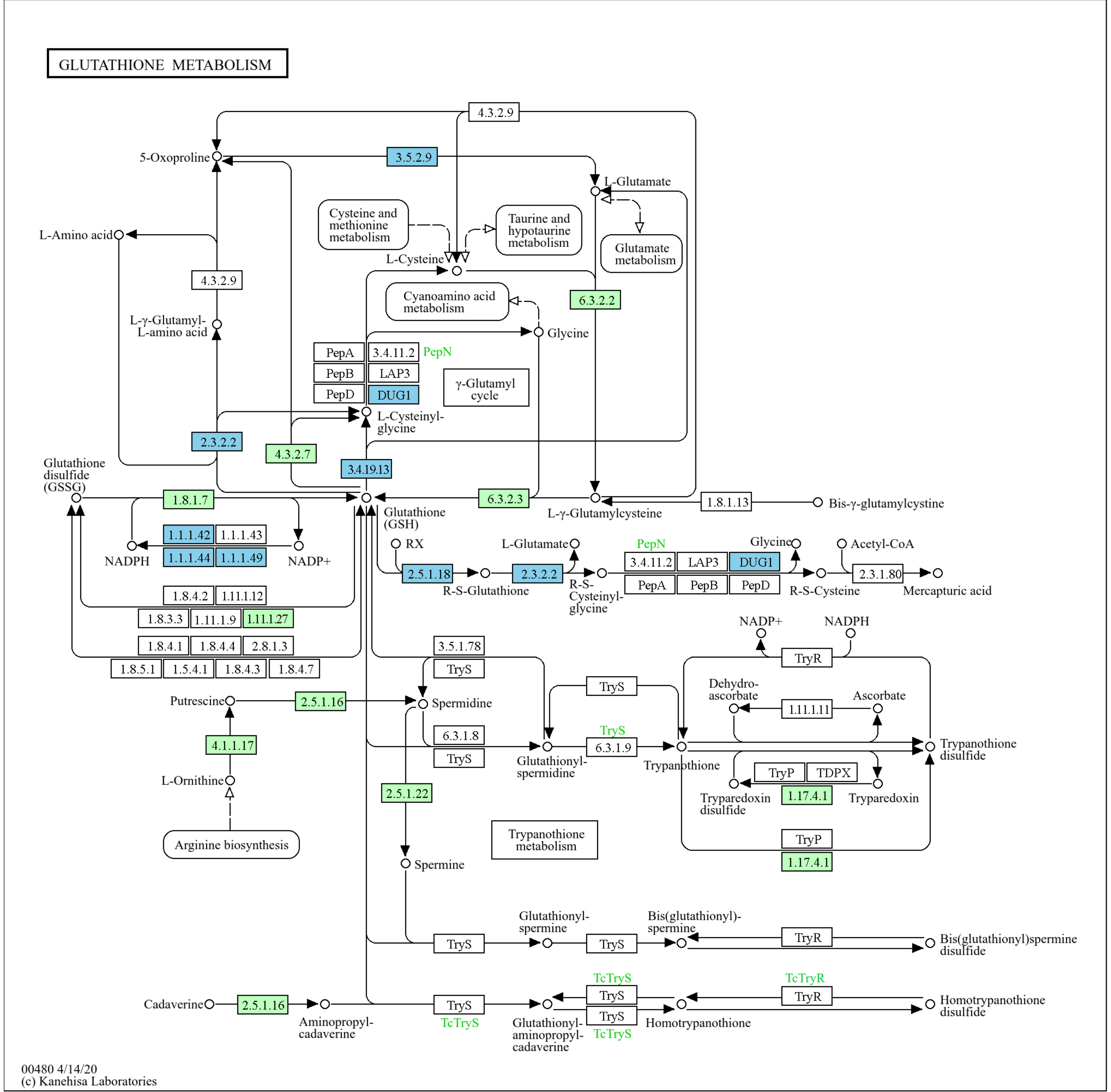
GLUTATHIONE METABOLISM
4.3.2.9
5-Oxoproline
3.5.2.9
L-Glutamate
Cysteine and methionine metabolism
Taurine and hypotaurine metabolism
Glutamate metabolism
L-Amino acid
4.3.2.9
L-Cysteine
Cyanoamino acid metabolism
6.3.2.2
L-γ-Glutamyl-L-amino acid
Glycine
PepA
3.4.11.2
PepN
PepB
LAP3
PepD
DUG1
γ-Glutamyl cycle
L-Cysteinyl-glycine
2.3.2.2
4.3.2.7
Glutathione disulfide (GSSG)
3.4.19.13
1.8.1.7
6.3.2.3
Glutathione (GSH)
L-γ-Glutamylcysteine
1.8.1.13
Bis-γ-glutamylcystine
1.1.1.42
1.1.1.43
NADPH
1.1.1.44
1.1.1.49
NADP+
RX
L-Glutamate
PepN
Glycine
Acetyl-CoA
3.4.11.2
LAP3
DUG1
2.5.1.18
2.3.2.2
2.3.1.80
PepA
PepB
PepD
R-S-Glutathione
R-S-Cysteinyl-glycine
R-S-Cysteine
Mercapturic acid
1.8.4.2
1.11.1.12
1.8.3.3
1.11.1.9
1.11.1.27
NADP+
NADPH
1.8.4.1
1.8.4.4
2.8.1.3
3.5.1.78
TryR
1.8.5.1
1.5.4.1
1.8.4.3
1.8.4.7
TryS
TryS
Dehydro-ascorbate
Ascorbate
Putrescine
2.5.1.16
Spermidine
1.11.1.11
TryS
6.3.1.8
6.3.1.9
Trypanothione disulfide
4.1.1.17
TryS
Glutathionyl-spermidine
Trypanothione
TryP
TDPX
L-Ornithine
2.5.1.22
Tryparedoxin disulfide
1.17.4.1
Tryparedoxin
TryP
Trypanothione metabolism
1.17.4.1
Arginine biosynthesis
Spermine
Glutathionyl-spermine
Bis(glutathionyl)-spermine
TryR
Bis(glutathionyl)spermine disulfide
TryS
TryS
TcTryS
TcTryR
TryS
TryR
Cadaverine
2.5.1.16
TryS
Homotrypanothione disulfide
TryS
Aminopropyl-cadaverine
TcTryS
Glutathionyl-aminopropyl-cadaverine
TcTryS
Homotrypanothione
00480 4/14/20
(c) Kanehisa Laboratories

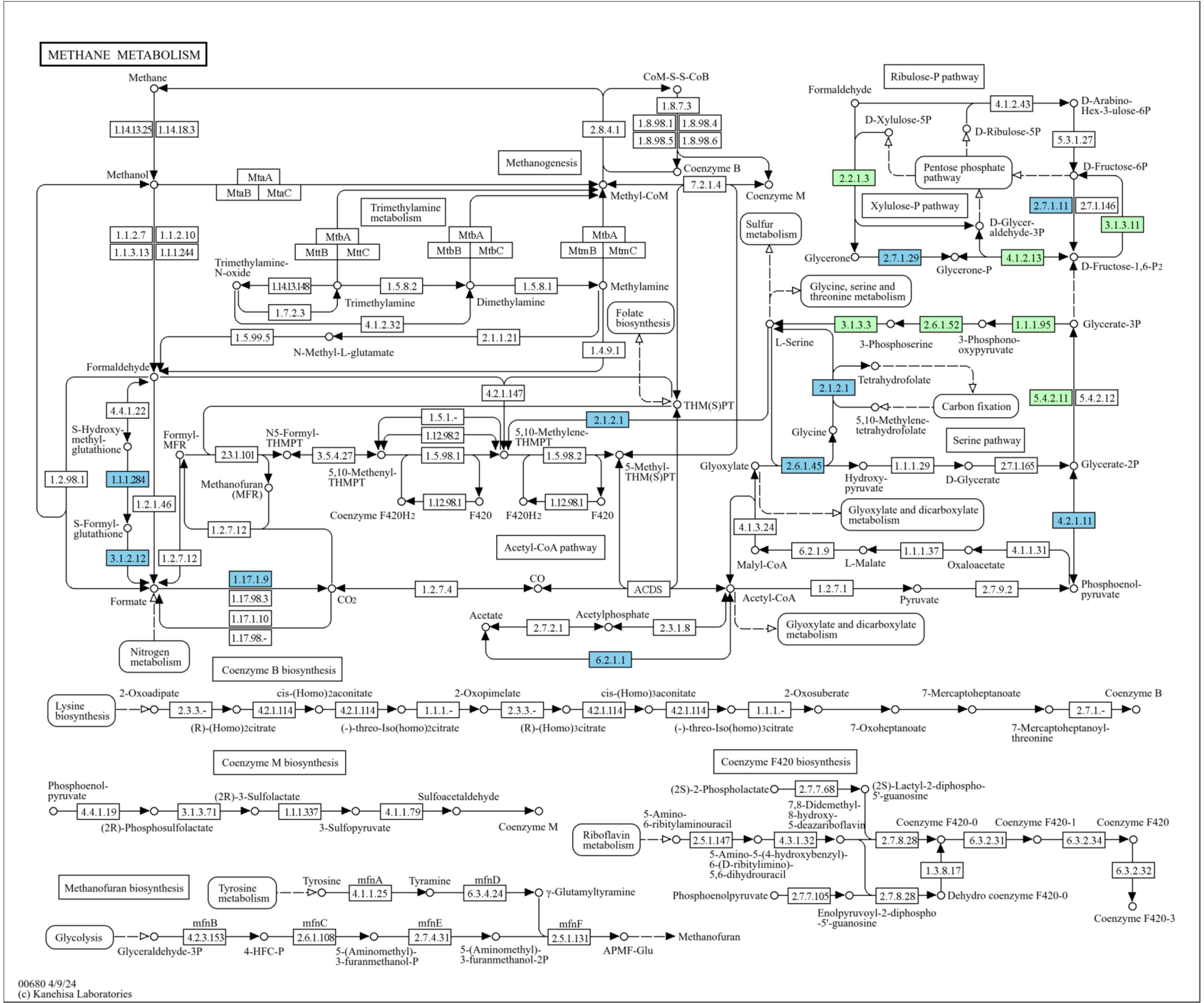
METHANE METABOLISM
Methane
CoM-S-S-CoB
Ribulose-P pathway
Formaldehyde
1.8.7.3
1.8.98.1
1.8.98.4
1.8.98.5
1.8.98.6
4.1.2.43
D-Arabino-Hex-3-ulose-6P
1.14.13.25
1.14.18.3
2.8.4.1
D-Xylulose-5P
D-Ribulose-5P
5.3.1.27
Methanogenesis
Coenzyme B
Methanol
MtaA
MtaB
MtaC
7.2.1.4
Methyl-CoM
Coenzyme M
2.2.1.3
Pentose phosphate pathway
D-Fructose-6P
Trimethylamine metabolism
Xylulose-P pathway
2.7.1.11
2.7.1.146
Sulfur metabolism
D-Glyceraldehyde-3P
3.1.3.11
1.1.2.7
1.1.2.10
MtbA
MttB
MttC
MtbA
MtbB
MtbC
MtbA
MtmB
MtmC
1.1.3.13
1.1.1.244
Glycerone
2.7.1.29
Glycerone-P
4.1.2.13
D-Fructose-1,6-P2
Trimethylamine-N-oxide
1.14.13.148
1.5.8.2
1.5.8.1
Methylamine
Trimethylamine
Dimethylamine
Glycine, serine and threonine metabolism
1.7.2.3
Folate biosynthesis
4.1.2.32
3.1.3.3
2.6.1.52
1.1.1.95
Glycerate-3P
1.5.99.5
2.1.1.21
L-Serine
3-Phosphoserine
3-Phosphonooxypyruvate
N-Methyl-L-glutamate
1.4.9.1
Formaldehyde
Tetrahydrofolate
2.1.2.1
4.4.1.22
4.2.1.147
Carbon fixation
5.4.2.11
5.4.2.12
THM(S)PT
S-Hydroxymethylglutathione
1.5.1.-
2.1.2.1
5,10-Methylenetetrahydrofolate
Formyl-MFR
N5-Formyl-THMPT
5,10-Methylene-THMPT
Glycine
Serine pathway
1.12.98.2
2.3.1.101
3.5.4.27
1.5.98.1
1.5.98.2
5-Methyl-THM(S)PT
Glyoxylate
2.6.1.45
1.1.1.29
2.7.1.165
Glycerate-2P
1.2.98.1
1.1.1.284
5,10-Methenyl-THMPT
Hydroxypyruvate
D-Glycerate
Methanofuran (MFR)
1.2.1.46
1.12.98.1
1.12.98.1
Glyoxylate and dicarboxylate metabolism
Coenzyme F420H2
F420
F420H2
F420
S-Formylglutathione
1.2.7.12
4.1.3.24
4.2.1.11
Acetyl-CoA pathway
6.2.1.9
1.1.1.37
4.1.1.31
3.1.2.12
1.2.7.12
Malyl-CoA
L-Malate
Oxaloacetate
1.17.1.9
CO
Phosphoenolpyruvate
1.2.7.4
ACDS
1.2.7.1
2.7.9.2
Formate
1.17.98.3
CO2
Acetyl-CoA
Pyruvate
1.17.1.10
Acetate
Acetylphosphate
2.7.2.1
2.3.1.8
Glyoxylate and dicarboxylate metabolism
1.17.98.-
Nitrogen metabolism
6.2.1.1
Coenzyme B biosynthesis
Lysine biosynthesis
2-Oxoadipate
cis-(Homo)2aconitate
2-Oxopimelate
cis-(Homo)3aconitate
2-Oxosuberate
7-Mercaptoheptanoate
Coenzyme B
2.3.3.-
4.2.1.114
4.2.1.114
1.1.1.-
2.3.3.-
4.2.1.114
4.2.1.114
1.1.1.-
2.7.1.-
(R)-(Homo)2citrate
(-)-threo-Iso(homo)2citrate
(R)-(Homo)3citrate
(-)-threo-Iso(homo)3citrate
7-Oxoheptanoate
7-Mercaptoheptanoylthreonine
Coenzyme M biosynthesis
Coenzyme F420 biosynthesis
Phosphoenolpyruvate
(2R)-3-Sulfolactate
Sulfoacetaldehyde
4.4.1.19
3.1.3.71
1.1.1.337
4.1.1.79
(2R)-Phosphosulfolactate
3-Sulfopyruvate
Coenzyme M
(2S)-2-Phospholactate
2.7.7.68
(2S)-Lactyl-2-diphospho-5'-guanosine
5-Amino-6-ribitylaminouracil
7,8-Didemethyl-8-hydroxy-5-deazariboflavin
Coenzyme F420-0
Coenzyme F420-1
Coenzyme F420
Riboflavin metabolism
2.5.1.147
4.3.1.32
2.7.8.28
6.3.2.31
6.3.2.34
5-Amino-5-(4-hydroxybenzyl)-6-(D-ribitylimino)-5,6-dihydrouracil
1.3.8.17
6.3.2.32
Methanofuran biosynthesis
Tyrosine metabolism
Tyrosine
mfnA
4.1.1.25
Tyramine
mfnD
6.3.4.24
γ-Glutamyltyramine
Phosphoenolpyruvate
2.7.7.105
2.7.8.28
Dehydro coenzyme F420-0
Enolpyruvoyl-2-diphospho-5'-guanosine
Coenzyme F420-3
Glycolysis
mfnB
4.2.3.153
mfnC
2.6.1.108
mfnE
2.7.4.31
mfnF
2.5.1.131
Methanofuran
Glyceraldehyde-3P
4-HFC-P
5-(Aminomethyl)-3-furanmethanol-P
5-(Aminomethyl)-3-furanmethanol-2P
APMF-Glu
00680 4/9/24
(c) Kanehisa Laboratories

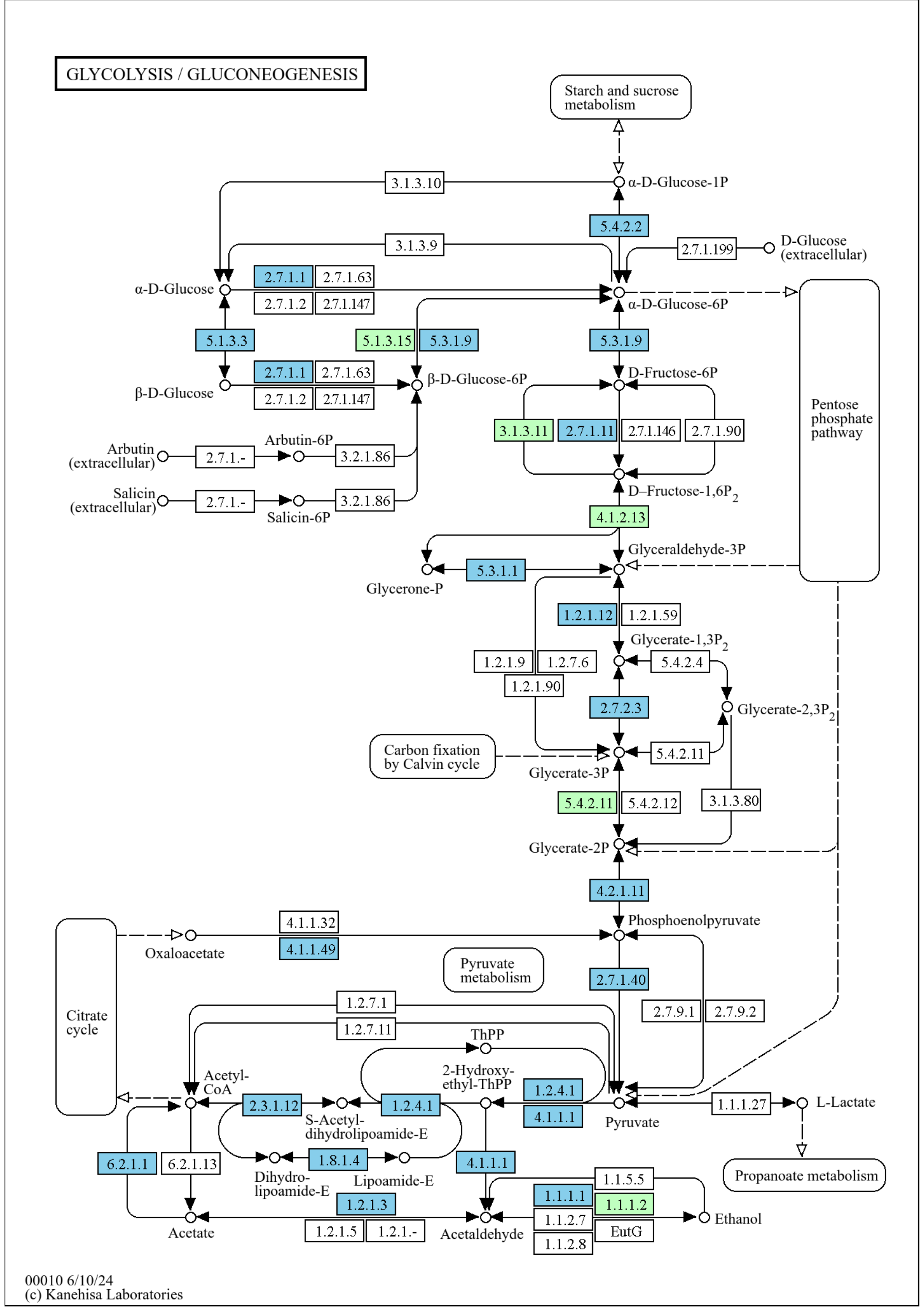
GLYCOLYSIS / GLUCONEOGENESIS
Starch and sucrose metabolism
3.1.3.10
α-D-Glucose-1P
5.4.2.2
3.1.3.9
2.7.1.199
D-Glucose (extracellular)
α-D-Glucose
2.7.1.1
2.7.1.63
2.7.1.2
2.7.1.147
α-D-Glucose-6P
5.1.3.3
5.1.3.15
5.3.1.9
5.3.1.9
2.7.1.1
2.7.1.63
2.7.1.2
2.7.1.147
β-D-Glucose
β-D-Glucose-6P
D-Fructose-6P
Pentose phosphate pathway
3.1.3.11
2.7.1.11
2.7.1.146
2.7.1.90
Arbutin (extracellular)
2.7.1.-
Arbutin-6P
3.2.1.86
Salicin (extracellular)
2.7.1.-
Salicin-6P
3.2.1.86
D–Fructose-1,6P2
4.1.2.13
Glyceraldehyde-3P
5.3.1.1
Glycerone-P
1.2.1.12
1.2.1.59
Glycerate-1,3P2
1.2.1.9
1.2.7.6
1.2.1.90
5.4.2.4
Glycerate-2,3P2
2.7.2.3
Carbon fixation by Calvin cycle
5.4.2.11
Glycerate-3P
5.4.2.11
5.4.2.12
3.1.3.80
Glycerate-2P
4.2.1.11
Phosphoenolpyruvate
4.1.1.32
4.1.1.49
Oxaloacetate
Citrate cycle
Pyruvate metabolism
2.7.1.40
2.7.9.1
2.7.9.2
1.2.7.1
1.2.7.11
ThPP
2-Hydroxy-ethyl-ThPP
Acetyl-CoA
2.3.1.12
S-Acetyl-dihydrolipoamide-E
1.2.4.1
1.2.4.1
4.1.1.1
Pyruvate
1.1.1.27
L-Lactate
Propanoate metabolism
6.2.1.1
6.2.1.13
Dihydro-lipoamide-E
1.8.1.4
Lipoamide-E
4.1.1.1
1.2.1.3
1.2.1.5
1.2.1.-
Acetate
Acetaldehyde
1.1.5.5
1.1.1.1
1.1.1.2
1.1.2.7
EutG
1.1.2.8
Ethanol
00010 6/10/24
(c) Kanehisa Laboratories

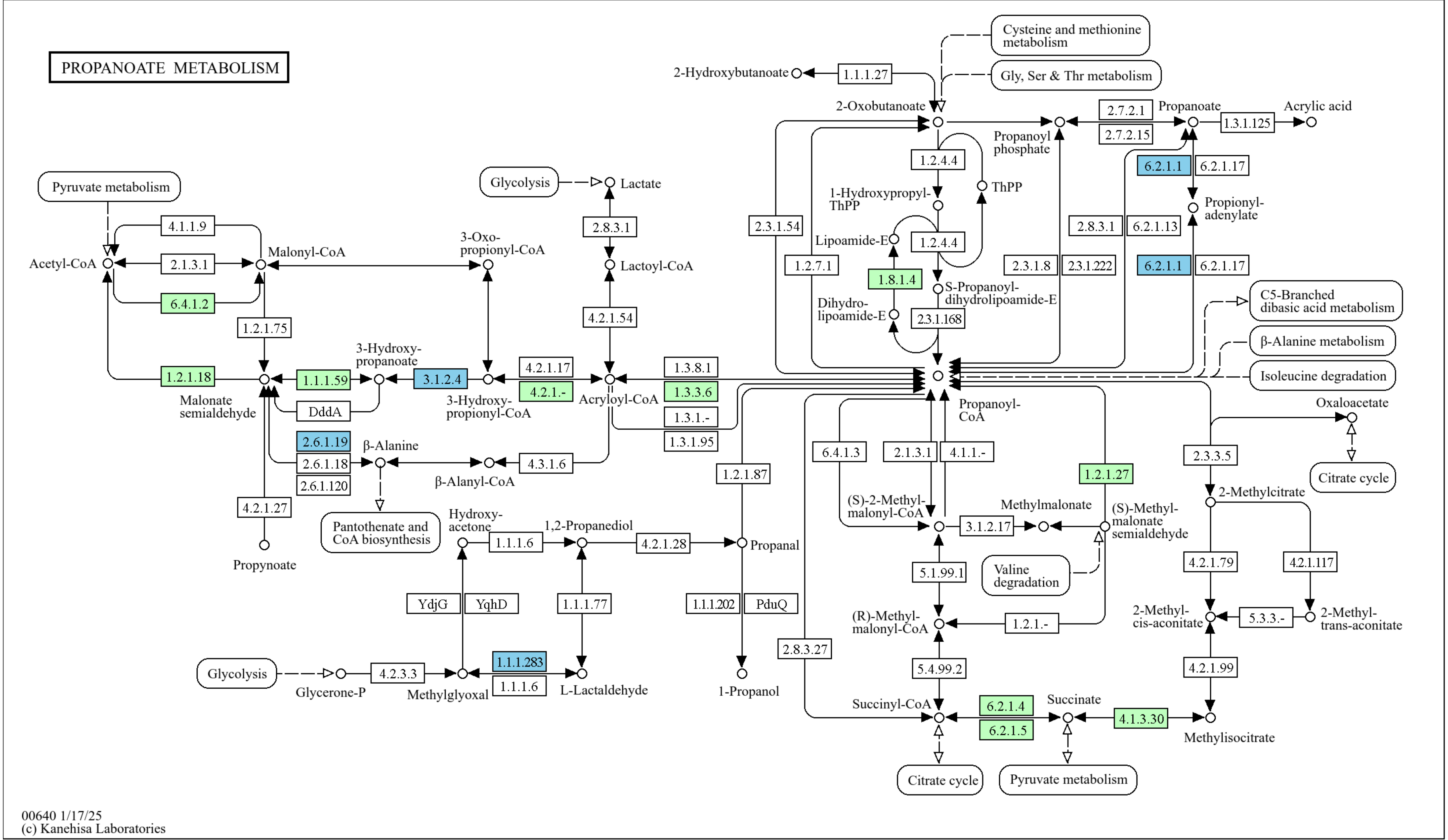
PROPANOATE METABOLISM
Cysteine and methionine metabolism
Gly, Ser & Thr metabolism
2-Hydroxybutanoate
1.1.1.27
2-Oxobutanoate
2.7.2.1
2.7.2.15
Propanoate
Acrylic acid
1.3.1.125
Propanoyl phosphate
1.2.4.4
ThPP
1-Hydroxypropyl-ThPP
6.2.1.1
6.2.1.17
Propionyl-adenylate
Pyruvate metabolism
Glycolysis
Lactate
2.8.3.1
Lactoyl-CoA
4.1.1.9
Acetyl-CoA
2.1.3.1
Malonyl-CoA
3-Oxo-propionyl-CoA
6.4.1.2
1.2.1.75
4.2.1.54
2.3.1.54
1.2.7.1
Lipoamide-E
1.8.1.4
Dihydro-lipoamide-E
S-Propanoyl-dihydrolipoamide-E
2.3.1.168
2.8.3.1
6.2.1.13
2.3.1.8
2.3.1.222
C5-Branched dibasic acid metabolism
β-Alanine metabolism
Isoleucine degradation
1.2.1.18
Malonate semialdehyde
1.1.1.59
DddA
3-Hydroxy-propanoate
3.1.2.4
3-Hydroxy-propionyl-CoA
4.2.1.17
4.2.1.-
Acryloyl-CoA
1.3.8.1
1.3.3.6
1.3.1.-
1.3.1.95
Propanoyl-CoA
Oxaloacetate
2.6.1.19
2.6.1.18
2.6.1.120
β-Alanine
β-Alanyl-CoA
4.3.1.6
1.2.1.87
6.4.1.3
2.1.3.1
4.1.1.-
1.2.1.27
2.3.3.5
Citrate cycle
2-Methylcitrate
4.2.1.27
Propynoate
Pantothenate and CoA biosynthesis
Hydroxy-acetone
1.1.1.6
1,2-Propanediol
4.2.1.28
Propanal
(S)-2-Methyl-malonyl-CoA
3.1.2.17
Methylmalonate
(S)-Methyl-malonate semialdehyde
Valine degradation
5.1.99.1
4.2.1.79
4.2.1.117
YdjG
YqhD
1.1.1.77
1.1.1.202
PduQ
(R)-Methyl-malonyl-CoA
1.2.1.-
2-Methyl-cis-aconitate
5.3.3.-
2-Methyl-trans-aconitate
2.8.3.27
5.4.99.2
4.2.1.99
Glycolysis
Glycerone-P
4.2.3.3
Methylglyoxal
1.1.1.283
1.1.1.6
L-Lactaldehyde
1-Propanol
Succinyl-CoA
6.2.1.4
6.2.1.5
Succinate
4.1.3.30
Methylisocitrate
Citrate cycle
Pyruvate metabolism
00640 1/17/25
(c) Kanehisa Laboratories

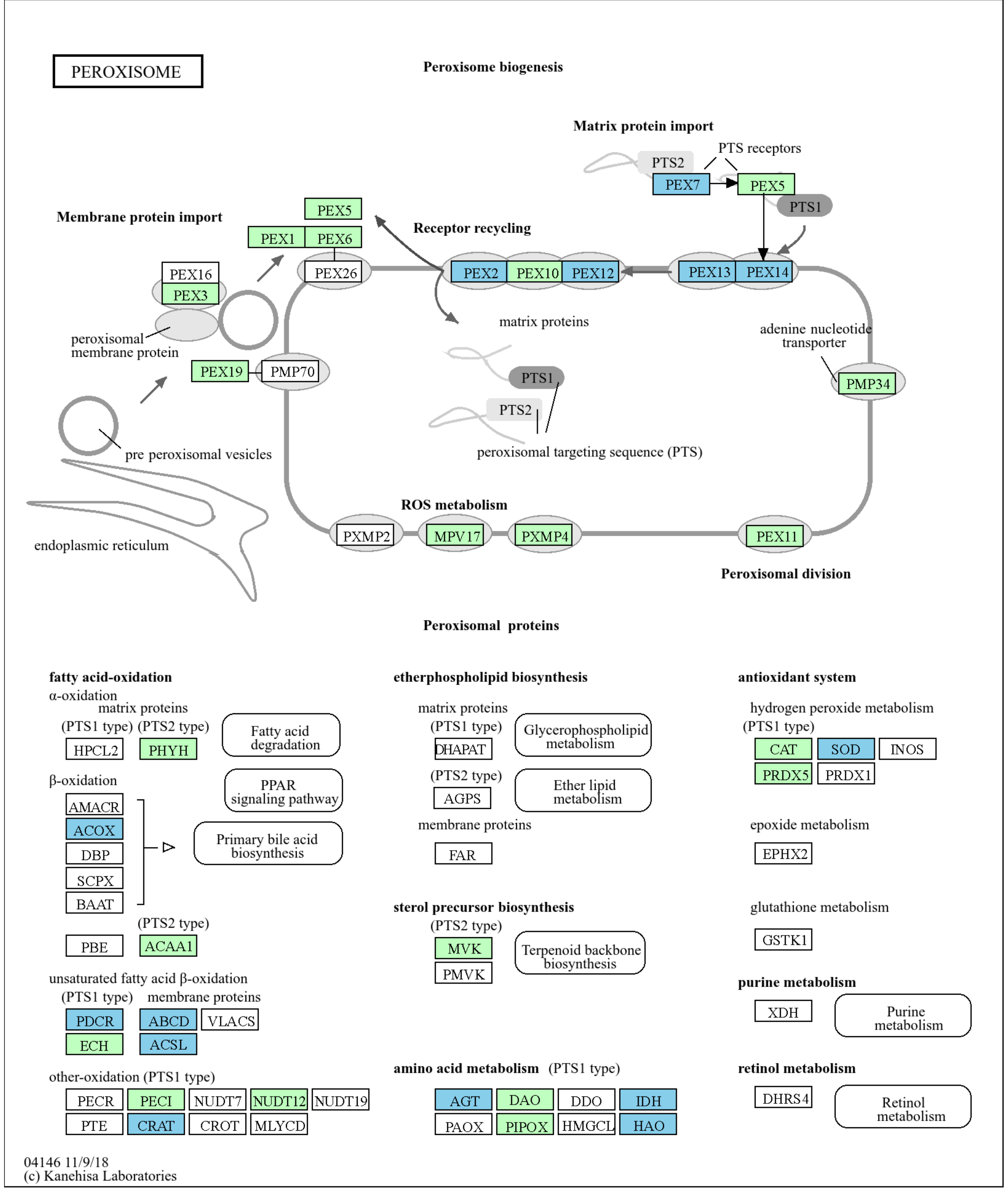
PEROXISOME
Peroxisome biogenesis
Matrix protein import
PTS2
PTS receptors
PEX7
PEX5
PTS1
Membrane protein import
PEX5
PEX1
PEX6
Receptor recycling
PEX16
PEX3
PEX26
PEX2
PEX10
PEX12
PEX13
PEX14
matrix proteins
peroxisomal membrane protein
adenine nucleotide transporter
PEX19
PMP70
PTS1
PMP34
PTS2
pre peroxisomal vesicles
peroxisomal targeting sequence (PTS)
ROS metabolism
endoplasmic reticulum
PXMP2
MPV17
PXMP4
PEX11
Peroxisomal division
Peroxisomal proteins
fatty acid-oxidation
α-oxidation
matrix proteins
(PTS1 type) (PTS2 type)
HPCL2
PHYH
Fatty acid degradation
β-oxidation
PPAR signaling pathway
AMACR
ACOX
DBP
SCPX
BAAT
Primary bile acid biosynthesis
(PTS2 type)
PBE
ACAA1
unsaturated fatty acid β-oxidation
(PTS1 type) membrane proteins
PDCR
ABCD
VLACS
ECH
ACSL
other-oxidation (PTS1 type)
PECR
PECI
NUDT7
NUDT12
NUDT19
PTE
CRAT
CROT
MLYCD
etherphospholipid biosynthesis
matrix proteins
(PTS1 type)
DHAPAT
Glycerophospholipid metabolism
(PTS2 type)
AGPS
Ether lipid metabolism
membrane proteins
FAR
sterol precursor biosynthesis
(PTS2 type)
MVK
PMVK
Terpenoid backbone biosynthesis
amino acid metabolism (PTS1 type)
AGT
DAO
DDO
IDH
PAOX
PIPOX
HMGCL
HAO
antioxidant system
hydrogen peroxide metabolism
(PTS1 type)
CAT
SOD
INOS
PRDX5
PRDX1
epoxide metabolism
EPHX2
glutathione metabolism
GSTK1
purine metabolism
XDH
Purine metabolism
retinol metabolism
DHRS4
Retinol metabolism
04146 11/9/18
(c) Kanehisa Laboratories

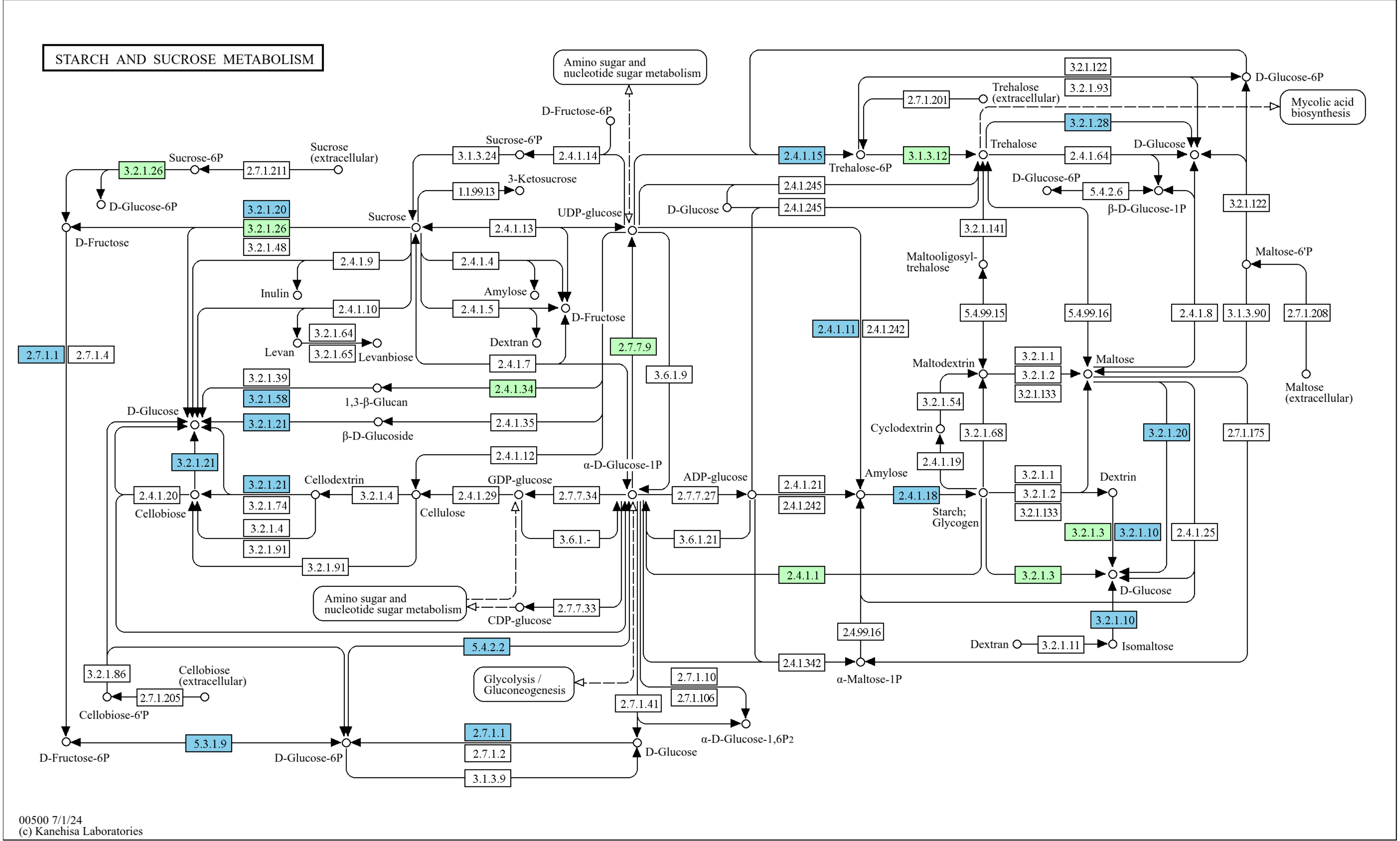
STARCH AND SUCROSE METABOLISM
Amino sugar and nucleotide sugar metabolism
D-Fructose-6P
Sucrose-6'P
3.1.3.24
2.4.1.14
Sucrose-6P
Sucrose (extracellular)
3.2.1.26
2.7.1.211
D-Glucose-6P
3-Ketosucrose
1.1.99.13
3.2.1.20
3.2.1.26
3.2.1.48
Sucrose
2.4.1.13
UDP-glucose
D-Fructose
2.4.1.9
2.4.1.4
Inulin
Amylose
2.4.1.10
2.4.1.5
D-Fructose
3.2.1.64
3.2.1.65
Levan
Levanbiose
Dextran
2.7.1.1
2.7.1.4
2.4.1.7
2.7.7.9
3.6.1.9
3.2.1.39
3.2.1.58
2.4.1.34
1,3-β-Glucan
D-Glucose
3.2.1.21
2.4.1.35
β-D-Glucoside
3.2.1.21
2.4.1.12
α-D-Glucose-1P
3.2.1.21
Cellodextrin
GDP-glucose
ADP-glucose
2.4.1.20
3.2.1.74
3.2.1.4
2.4.1.29
2.7.7.34
2.7.7.27
Cellobiose
Cellulose
3.2.1.4
3.2.1.91
3.6.1.-
3.6.1.21
3.2.1.91
Amino sugar and nucleotide sugar metabolism
2.7.7.33
CDP-glucose
5.4.2.2
3.2.1.86
Cellobiose (extracellular)
2.7.1.205
Cellobiose-6'P
Glycolysis / Gluconeogenesis
2.7.1.10
2.7.1.106
2.7.1.41
D-Fructose-6P
5.3.1.9
D-Glucose-6P
2.7.1.1
2.7.1.2
D-Glucose
α-D-Glucose-1,6P2
3.1.3.9
3.2.1.122
D-Glucose-6P
Trehalose (extracellular)
3.2.1.93
2.7.1.201
Mycolic acid biosynthesis
3.2.1.28
2.4.1.15
3.1.3.12
Trehalose
D-Glucose
Trehalose-6P
2.4.1.64
2.4.1.245
D-Glucose-6P
5.4.2.6
D-Glucose
2.4.1.245
β-D-Glucose-1P
3.2.1.122
3.2.1.141
Maltooligosyl-trehalose
Maltose-6'P
2.4.1.11
2.4.1.242
5.4.99.15
5.4.99.16
2.4.1.8
3.1.3.90
2.7.1.208
3.2.1.1
Maltodextrin
3.2.1.2
Maltose
3.2.1.133
Maltose (extracellular)
3.2.1.54
Cyclodextrin
3.2.1.68
3.2.1.20
2.7.1.175
2.4.1.19
Amylose
3.2.1.1
Dextrin
2.4.1.21
2.4.1.242
2.4.1.18
3.2.1.2
3.2.1.133
Starch; Glycogen
3.2.1.3
3.2.1.10
2.4.1.25
2.4.1.1
3.2.1.3
D-Glucose
3.2.1.10
2.4.99.16
Dextran
3.2.1.11
Isomaltose
2.4.1.342
α-Maltose-1P
00500 7/1/24
(c) Kanehisa Laboratories

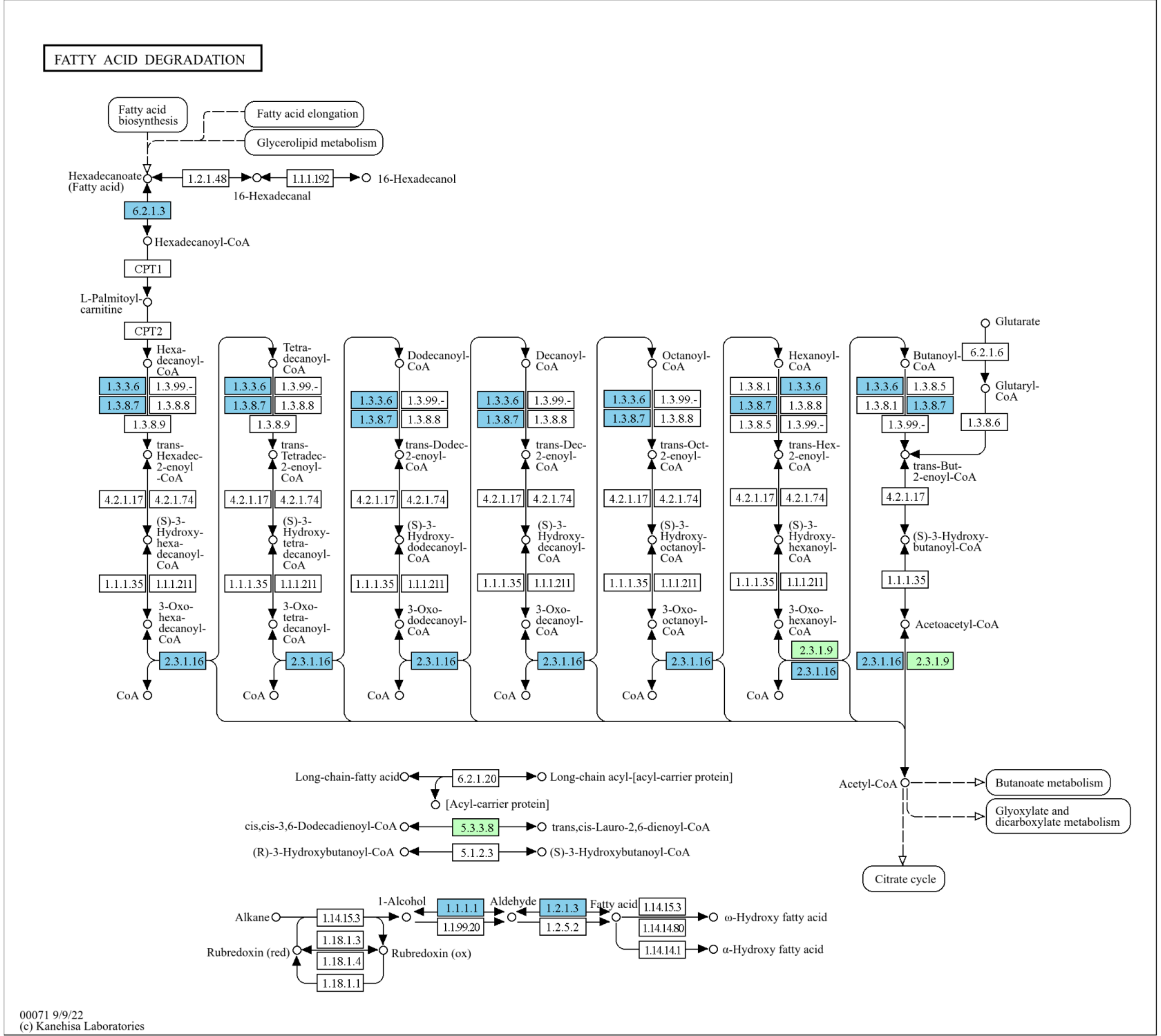
FATTY ACID DEGRADATION
Fatty acid biosynthesis
Fatty acid elongation
Glycerolipid metabolism
Hexadecanoate (Fatty acid)
1.2.1.48
16-Hexadecanal
1.1.1.192
16-Hexadecanol
6.2.1.3
Hexadecanoyl-CoA
CPT1
L-Palmitoyl-carnitine
CPT2
Hexa-decanoyl-CoA
Tetra-decanoyl-CoA
Dodecanoyl-CoA
Decanoyl-CoA
Octanoyl-CoA
Hexanoyl-CoA
Butanoyl-CoA
Glutarate
6.2.1.6
Glutaryl-CoA
1.3.8.6
1.3.3.6
1.3.99.-
1.3.8.7
1.3.8.8
1.3.8.9
1.3.8.1
1.3.8.5
trans-Hexadec-2-enoyl-CoA
trans-Tetradec-2-enoyl-CoA
trans-Dodec-2-enoyl-CoA
trans-Dec-2-enoyl-CoA
trans-Oct-2-enoyl-CoA
trans-Hex-2-enoyl-CoA
trans-But-2-enoyl-CoA
4.2.1.17
4.2.1.74
(S)-3-Hydroxy-hexa-decanoyl-CoA
(S)-3-Hydroxy-tetra-decanoyl-CoA
(S)-3-Hydroxy-dodecanoyl-CoA
(S)-3-Hydroxy-decanoyl-CoA
(S)-3-Hydroxy-octanoyl-CoA
(S)-3-Hydroxy-hexanoyl-CoA
(S)-3-Hydroxy-butanoyl-CoA
1.1.1.35
1.1.1.211
3-Oxo-hexa-decanoyl-CoA
3-Oxo-tetra-decanoyl-CoA
3-Oxo-dodecanoyl-CoA
3-Oxo-decanoyl-CoA
3-Oxo-octanoyl-CoA
3-Oxo-hexanoyl-CoA
Acetoacetyl-CoA
2.3.1.16
2.3.1.9
CoA
Acetyl-CoA
Butanoate metabolism
Glyoxylate and dicarboxylate metabolism
Citrate cycle
Long-chain-fatty acid
6.2.1.20
Long-chain acyl-[acyl-carrier protein]
[Acyl-carrier protein]
cis,cis-3,6-Dodecadienoyl-CoA
5.3.3.8
trans,cis-Lauro-2,6-dienoyl-CoA
(R)-3-Hydroxybutanoyl-CoA
5.1.2.3
(S)-3-Hydroxybutanoyl-CoA
Alkane
1.14.15.3
1.18.1.3
1.18.1.4
1.18.1.1
Rubredoxin (red)
Rubredoxin (ox)
1-Alcohol
1.1.1.1
1.1.99.20
Aldehyde
1.2.1.3
1.2.5.2
Fatty acid
1.14.15.3
1.14.14.80
1.14.14.1
ω-Hydroxy fatty acid
α-Hydroxy fatty acid
00071 9/9/22
(c) Kanehisa Laboratories

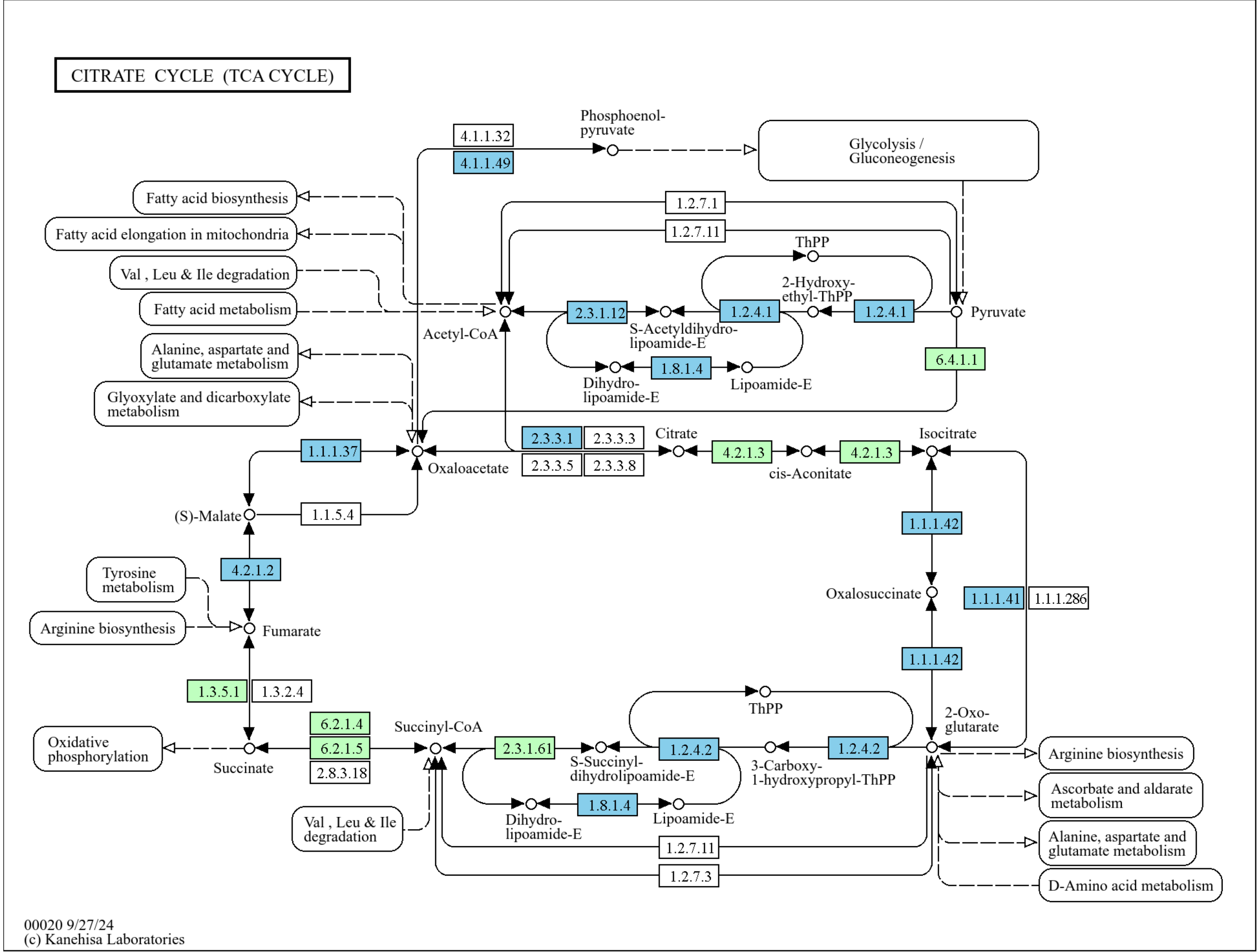
CITRATE CYCLE (TCA CYCLE)
4.1.1.32
4.1.1.49
Phosphoenol-pyruvate
Glycolysis / Gluconeogenesis
Fatty acid biosynthesis
Fatty acid elongation in mitochondria
Val , Leu & Ile degradation
Fatty acid metabolism
1.2.7.1
1.2.7.11
ThPP
2-Hydroxy-ethyl-ThPP
2.3.1.12
1.2.4.1
1.2.4.1
Pyruvate
Acetyl-CoA
S-Acetyldihydro-lipoamide-E
1.8.1.4
Dihydro-lipoamide-E
Lipoamide-E
6.4.1.1
Alanine, aspartate and glutamate metabolism
Glyoxylate and dicarboxylate metabolism
2.3.3.1
2.3.3.3
2.3.3.5
2.3.3.8
Citrate
4.2.1.3
4.2.1.3
cis-Aconitate
Isocitrate
1.1.1.37
Oxaloacetate
(S)-Malate
1.1.5.4
4.2.1.2
1.1.1.42
Tyrosine metabolism
Arginine biosynthesis
Fumarate
Oxalosuccinate
1.1.1.41
1.1.1.286
1.1.1.42
1.3.5.1
1.3.2.4
ThPP
6.2.1.4
6.2.1.5
2.8.3.18
Succinyl-CoA
2-Oxo-glutarate
Oxidative phosphorylation
Succinate
2.3.1.61
S-Succinyl-dihydrolipoamide-E
1.2.4.2
3-Carboxy-1-hydroxypropyl-ThPP
1.2.4.2
Arginine biosynthesis
Ascorbate and aldarate metabolism
Dihydro-lipoamide-E
1.8.1.4
Lipoamide-E
Val , Leu & Ile degradation
1.2.7.11
1.2.7.3
Alanine, aspartate and glutamate metabolism
D-Amino acid metabolism
00020 9/27/24
(c) Kanehisa Laboratories

**Supplementary Figure 3.** Predicted pathways associated with BaP degradation (YNB + BaP) and stress response in *Debaryomyces hansenii*. Enzymes absent in the genome are shown in white, enzymes present but not expressed are indicated in green, and enzymes overexpressed in BaP-treated cultures are shown in blue.
