# Supplementary material for: Transcriptomic profiling of Debaryomyces hansenii reveals detoxification and stress responses to benzo(a)pyrene exposure
Source: Appl Environ Microbiol. 2025 Sep 16;91(10):e01557-25. doi: 10.1128/aem.01557-25 (PMC12542653; doi:10.1128/aem.01557-25)
Supplement: Figure S4 — Predicted metabolic pathways associated with energy metabolism and redox balance in Debaryomyces hansenii under YNBG + BaP treatment. [file aem.01557-25-s0004.pdf]

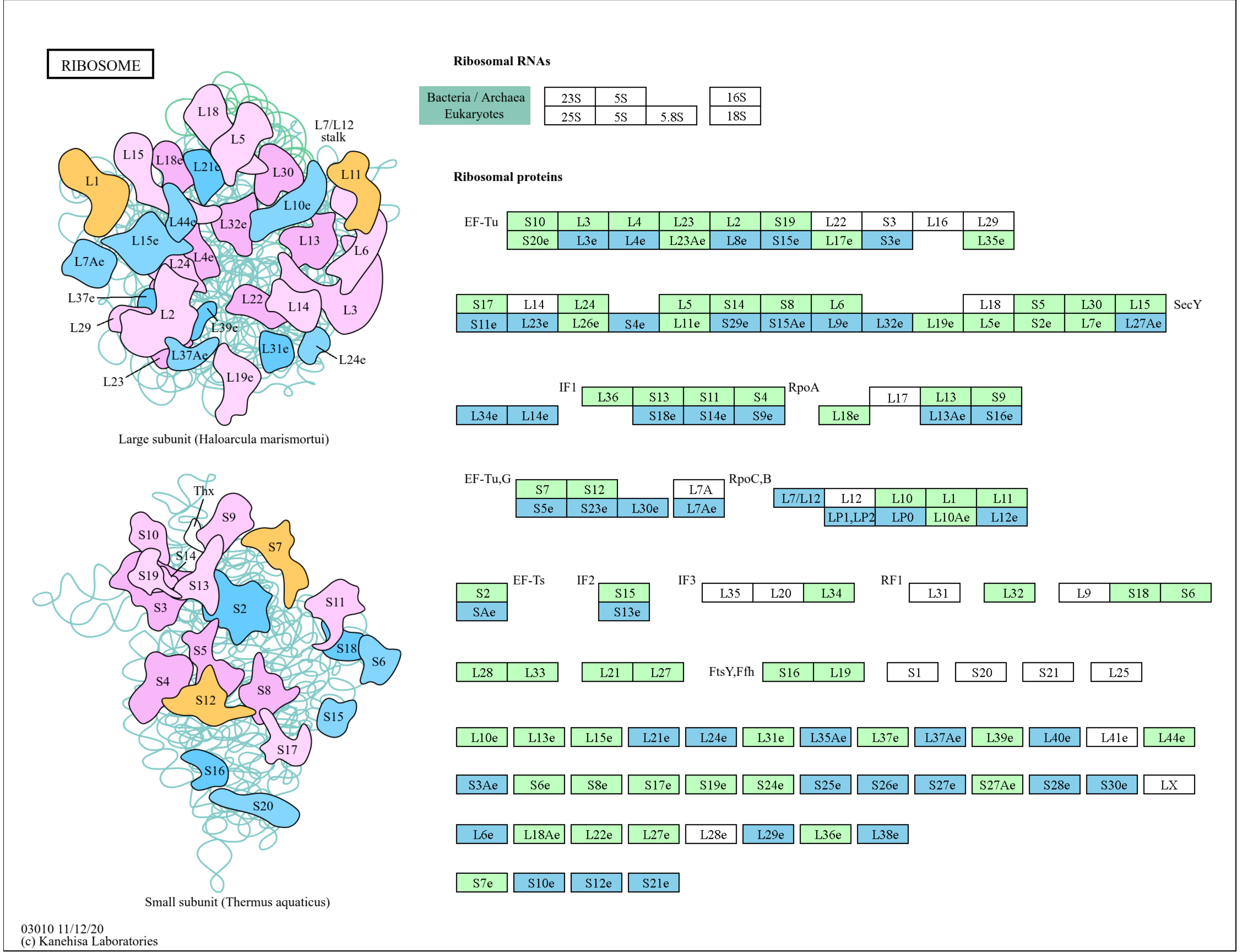

In all figures, white indicates enzymes absent in *Debaryomyces hansenii*, green indicates those present but not expressed, and blue indicates those that were overexpressed.

OXIDATIVE PHOSPHORYLATION

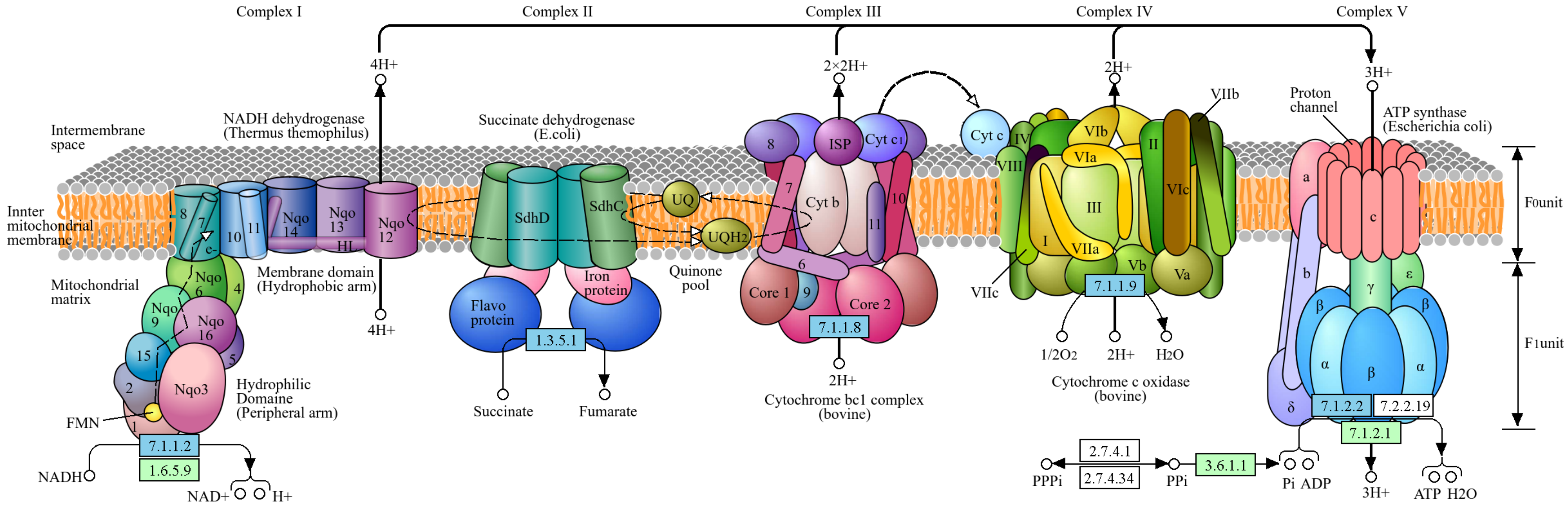

NADH dehydrogenase

E	ND1	ND2	ND3	ND4	ND4L	ND5	ND6										
E	Ndufs1	Ndufs2	Ndufs3	Ndufs4	Ndufs5	Ndufs6	Ndufs7	Ndufs8	Ndufv1	Ndufv2	Ndufv3						
B/A	NuoA	NuoB	NuoC	NuoD	NuoE	NuoF	NuoG	NuoH	NuoI	NuoJ	NuoK	NuoL	NuoM	NuoN			
E/B	NdhC	NdhK	NdhJ	NdhH	NdhA	NdhI	NdhG	NdhE	NdhF	NdhD	NdhB	NdhL	NdhM	NdhN	HoxE	HoxF	HoxU
E	Ndufa1	Ndufa2	Ndufa3	Ndufa4	Ndufa5	Ndufa6	Ndufa7	Ndufa8	Ndufa9	Ndufa10	Ndufab1	Ndufa11	Ndufa12	Ndufa13			
E	Ndufb1	Ndufb2	Ndufb3	Ndufb4	Ndufb5	Ndufb6	Ndufb7	Ndufb8	Ndufb9	Ndufb10	Ndufb11	Ndufc1	Ndufc2				

Succinate dehydrogenase / Fumarate reductase

E	SDHC	SDHD	SDHA	SDHB	
B/A	SdhC	SdhD	SdhA	SdhB	
			FrdA	FrdB	FrdC	FrdD

Cytochrome c reductase

E/B/A	ISP	Cyt b	Cyt 1				
E	COR1	QCR2	QCR6	QCR7	QCR8	QCR9	QCR10

Cytochrome c oxidase

E	COX10	COX3	COX1	COX2	COX4	COX5A	COX5B	COX6A	COX6B	COX6C	COX7A	COX7B	COX7C	COX8		COX17
E/B/A															COX11	COX15
B/A	CyoE	CyoD	CyoC	CyoB	CyoA											
		CoxD	CoxC	CoxA	CoxB											
		QoxD	QoxC	QoxB	QoxA											
		SoxD	SoxC	SoxB	SoxA											

Cytochrome c oxidase , cbb3-type

B	I	II	IV	III

Cytochrome bd complex

B/A	CydA	CydB	CydX

Cytochrome c

CYC

F-type ATPase (Bacteria)

alpha	beta	gamma	delta	epsilon
a	b	c		

F-type ATPase (Eukaryotes)

alpha	beta	gamma	delta	epsilon	
OSCP	a	b	c	d	e
f	g	f6/h	j	k	8

V/A-type ATPase (Bacteria, Archaea)

A	B	C	D	E	F	G/H
I	K					

V-type ATPase (Eukaryotes)

A	B	C	D	E	F	G	H
a	c	d	e	S1			

00190 12/15/23

(c) Kanehisa Laboratories

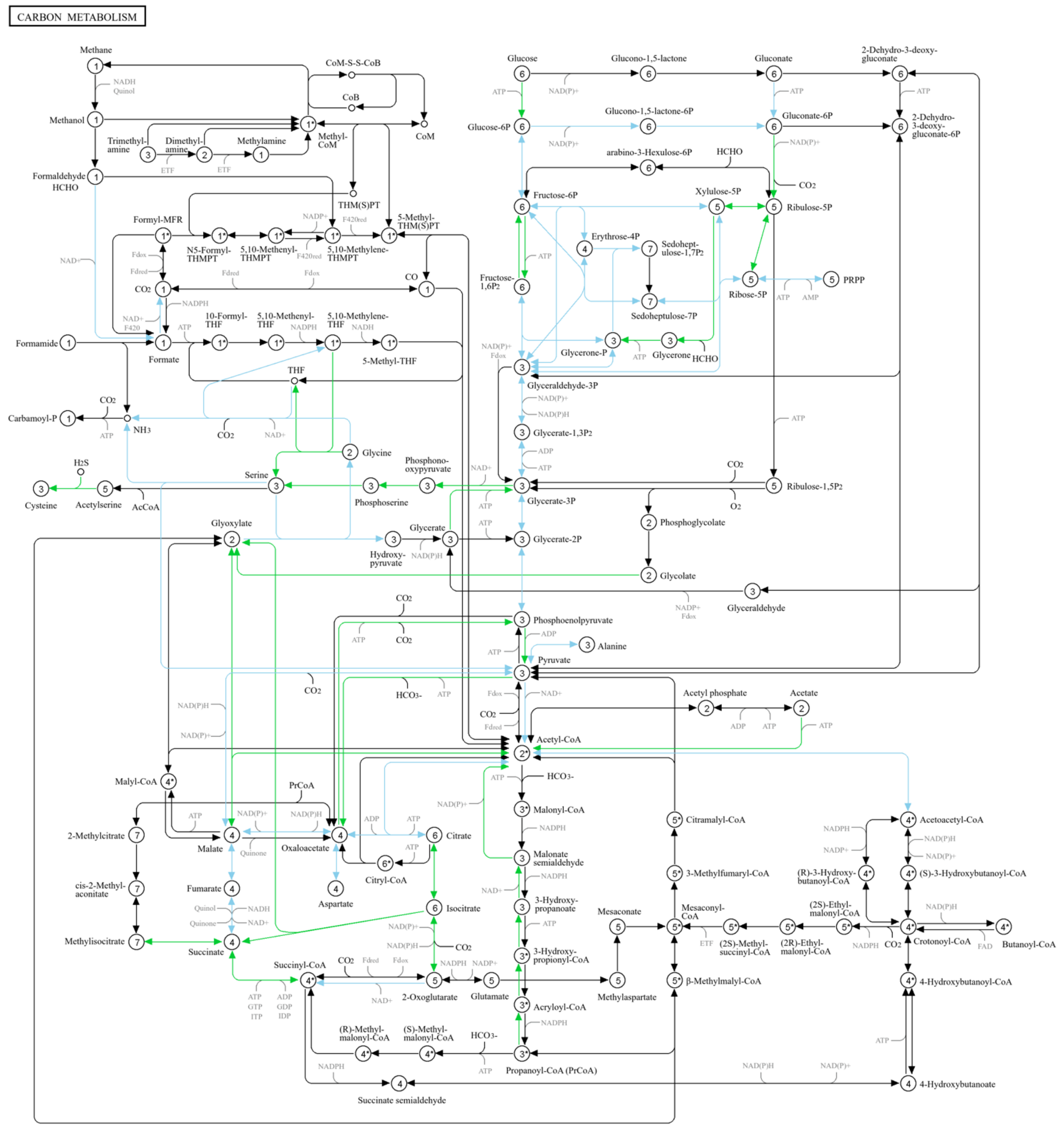

01200 4/24/25
(c) Kanehisa Laboratories

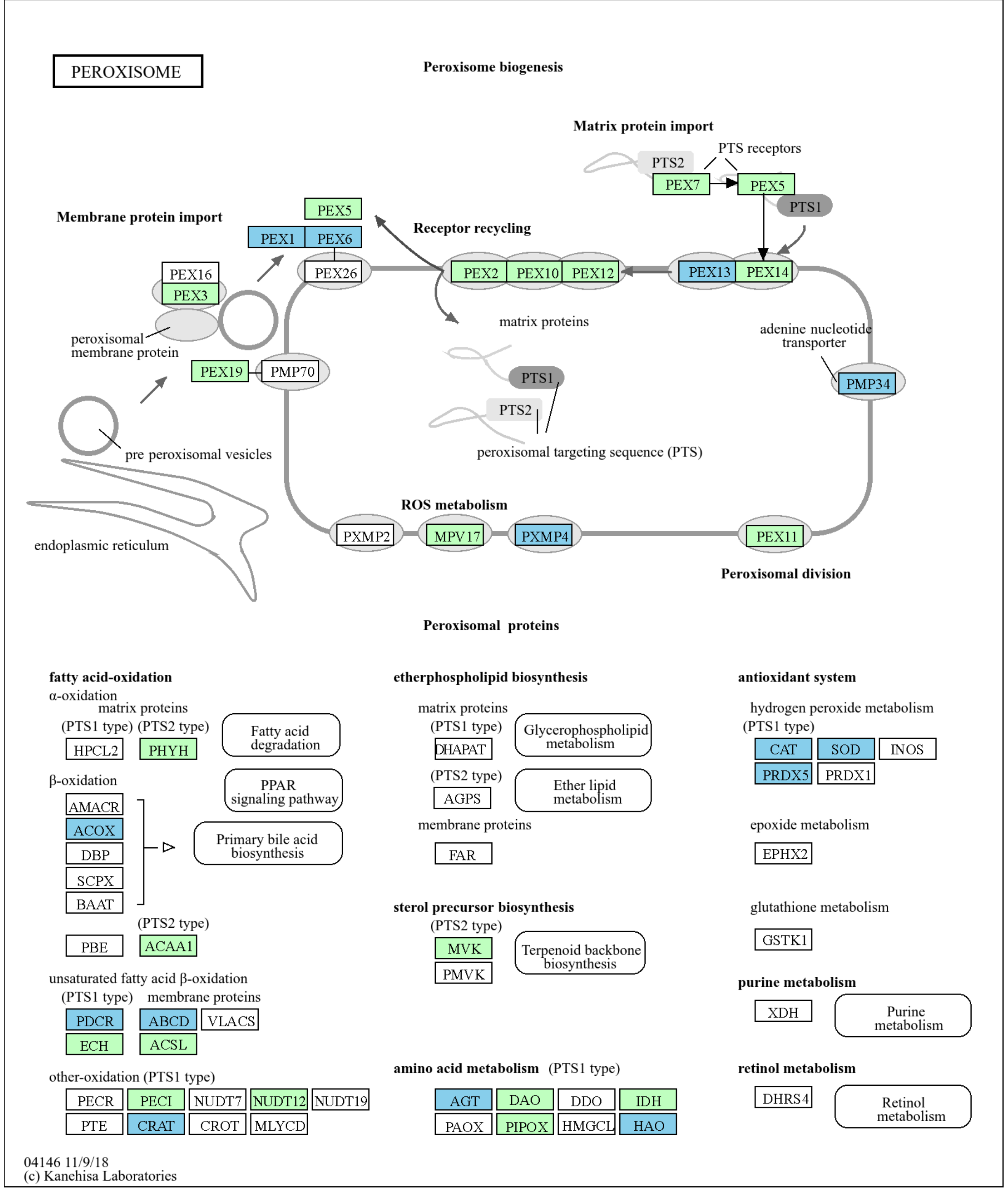
PEROXISOME
Peroxisome biogenesis
Matrix protein import
PTS receptors
PTS2
PEX7
PEX5
PTS1
Membrane protein import
PEX5
PEX1
PEX6
Receptor recycling
PEX16
PEX3
PEX26
PEX2
PEX10
PEX12
PEX13
PEX14
matrix proteins
peroxisomal
membrane protein
adenine nucleotide
transporter
PEX19
PMP70
PTS1
PMP34
PTS2
pre peroxisomal vesicles
peroxisomal targeting sequence (PTS)
ROS metabolism
endoplasmic reticulum
PXMP2
MPV17
PXMP4
PEX11
Peroxisomal division
Peroxisomal proteins
fatty acid-oxidation
α-oxidation
matrix proteins
(PTS1 type) (PTS2 type)
HPCL2
PHYH
Fatty acid degradation
β-oxidation
PPAR signaling pathway
AMACR
ACOX
DBP
SCPX
BAAT
Primary bile acid biosynthesis
(PTS2 type)
PBE
ACAA1
unsaturated fatty acid β-oxidation
(PTS1 type) membrane proteins
PDCR
ABCD
VLACS
ECH
ACSL
other-oxidation (PTS1 type)
PECR
PECI
NUDT7
NUDT12
NUDT19
PTE
CRAT
CROT
MLYCD
etherphospholipid biosynthesis
matrix proteins
(PTS1 type)
DHAPAT
Glycerophospholipid metabolism
(PTS2 type)
AGPS
Ether lipid metabolism
membrane proteins
FAR
sterol precursor biosynthesis
(PTS2 type)
MVK
PMVK
Terpenoid backbone biosynthesis
amino acid metabolism (PTS1 type)
AGT
DAO
DDO
IDH
PAOX
PIPOX
HMGCL
HAO
antioxidant system
hydrogen peroxide metabolism
(PTS1 type)
CAT
SOD
INOS
PRDX5
PRDX1
epoxide metabolism
EPHX2
glutathione metabolism
GSTK1
purine metabolism
XDH
Purine metabolism
retinol metabolism
DHRS4
Retinol metabolism
04146 11/9/18
(c) Kanehisa Laboratories

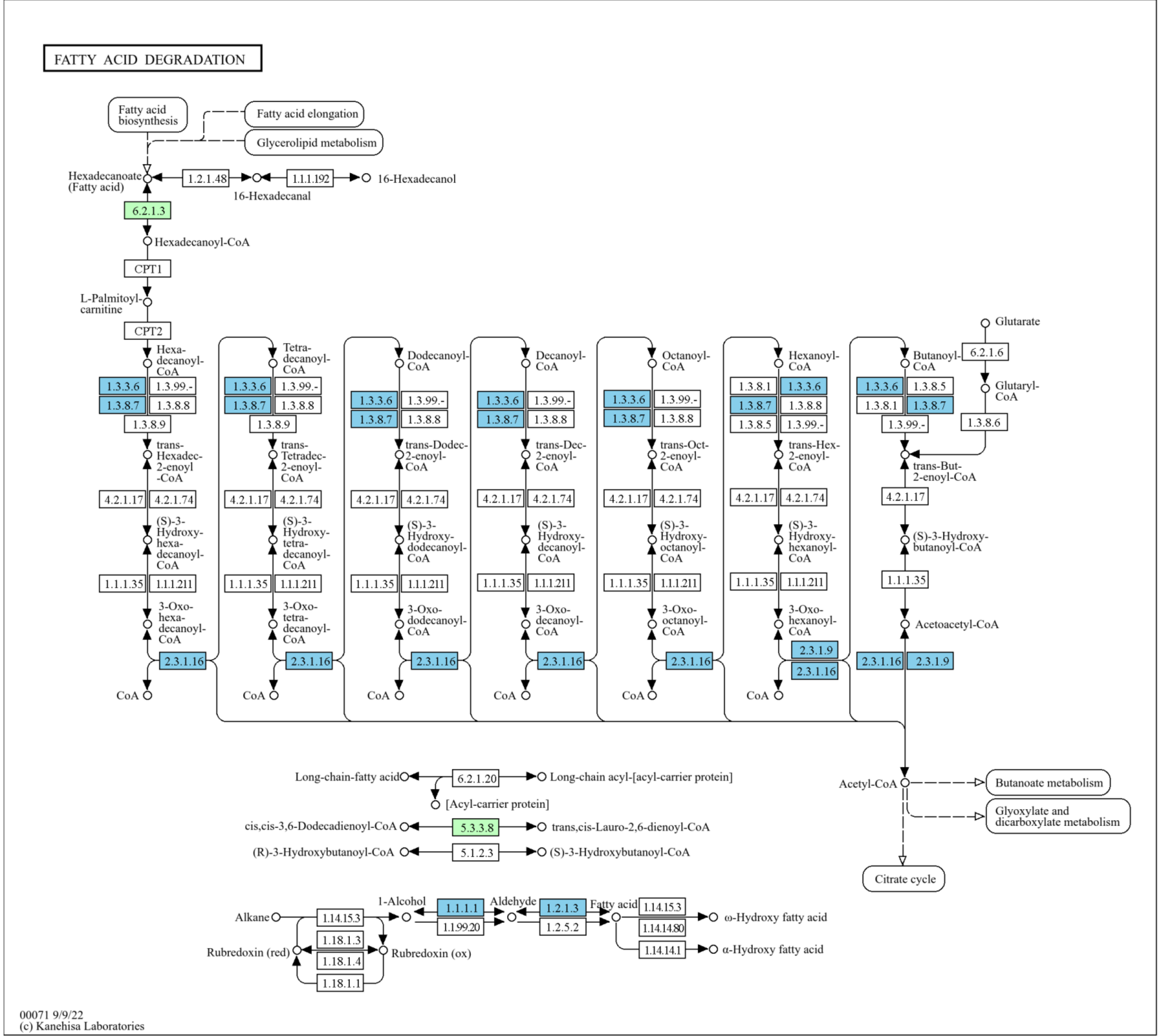
FATTY ACID DEGRADATION
Fatty acid biosynthesis
Fatty acid elongation
Glycerolipid metabolism
Hexadecanoate (Fatty acid)
1.2.1.48
1.1.1.192
16-Hexadecanol
16-Hexadecanal
6.2.1.3
Hexadecanoyl-CoA
CPT1
L-Palmitoyl-carnitine
CPT2
Hexa-decanoyl-CoA
Tetra-decanoyl-CoA
Dodecanoyl-CoA
Decanoyl-CoA
Octanoyl-CoA
Hexanoyl-CoA
Butanoyl-CoA
Glutarate
6.2.1.6
Glutaryl-CoA
1.3.8.6
1.3.3.6
1.3.99.-
1.3.8.7
1.3.8.8
1.3.8.9
1.3.8.1
1.3.8.5
trans-Hexadec-2-enoyl-CoA
trans-Tetradec-2-enoyl-CoA
trans-Dodec-2-enoyl-CoA
trans-Dec-2-enoyl-CoA
trans-Oct-2-enoyl-CoA
trans-Hex-2-enoyl-CoA
trans-But-2-enoyl-CoA
4.2.1.17
4.2.1.74
(S)-3-Hydroxy-hexa-decanoyl-CoA
(S)-3-Hydroxy-tetra-decanoyl-CoA
(S)-3-Hydroxy-dodecanoyl-CoA
(S)-3-Hydroxy-decanoyl-CoA
(S)-3-Hydroxy-octanoyl-CoA
(S)-3-Hydroxy-hexanoyl-CoA
(S)-3-Hydroxy-butanoyl-CoA
1.1.1.35
1.1.1.211
3-Oxo-hexa-decanoyl-CoA
3-Oxo-tetra-decanoyl-CoA
3-Oxo-dodecanoyl-CoA
3-Oxo-decanoyl-CoA
3-Oxo-octanoyl-CoA
3-Oxo-hexanoyl-CoA
Acetoacetyl-CoA
2.3.1.16
2.3.1.9
CoA
Acetyl-CoA
Butanoate metabolism
Glyoxylate and dicarboxylate metabolism
Citrate cycle
Long-chain-fatty acid
6.2.1.20
Long-chain acyl-[acyl-carrier protein]
[Acyl-carrier protein]
cis,cis-3,6-Dodecadienoyl-CoA
5.3.3.8
trans,cis-Lauro-2,6-dienoyl-CoA
(R)-3-Hydroxybutanoyl-CoA
5.1.2.3
(S)-3-Hydroxybutanoyl-CoA
Alkane
1.14.15.3
1-Alcohol
1.1.1.1
1.1.99.20
Aldehyde
1.2.1.3
1.2.5.2
Fatty acid
1.14.15.3
1.14.14.80
1.14.14.1
ω-Hydroxy fatty acid
α-Hydroxy fatty acid
1.18.1.3
1.18.1.4
1.18.1.1
Rubredoxin (red)
Rubredoxin (ox)
00071 9/9/22
(c) Kanehisa Laboratories

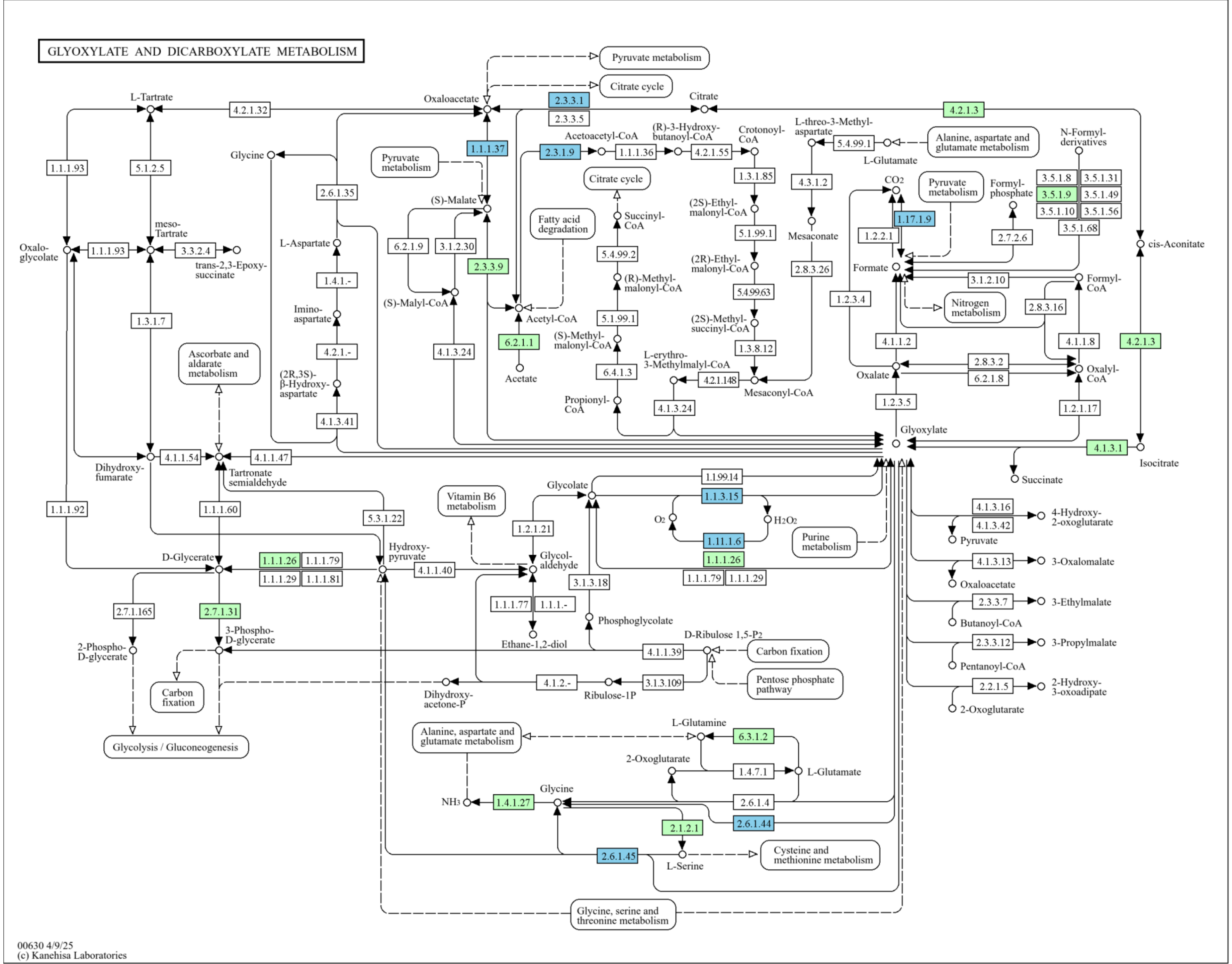
GLYOXYLATE AND DICARBOXYLATE METABOLISM
Pyruvate metabolism
Citrate cycle
L-Tartrate
4.2.1.32
Oxaloacetate
2.3.3.1
2.3.3.5
Citrate
4.2.1.3
Glycine
1.1.1.93
5.1.2.5
2.6.1.35
Pyruvate metabolism
1.1.1.37
2.3.1.9
Acetoacetyl-CoA
1.1.1.36
(R)-3-Hydroxy-butanoyl-CoA
4.2.1.55
Crotonoyl-CoA
L-threo-3-Methyl-aspartate
5.4.99.1
L-Glutamate
Alanine, aspartate and glutamate metabolism
N-Formyl-derivatives
Citrate cycle
1.3.1.85
4.3.1.2
CO2
Pyruvate metabolism
Formyl-phosphate
3.5.1.8
3.5.1.31
3.5.1.9
3.5.1.49
3.5.1.10
3.5.1.56
3.5.1.68
(S)-Malate
Fatty acid degradation
Succinyl-CoA
(2S)-Ethyl-malonyl-CoA
Mesaconate
1.17.1.9
1.2.2.1
2.7.2.6
meso-Tartrate
Oxalo-glycolate
1.1.1.93
3.3.2.4
trans-2,3-Epoxy-succinate
L-Aspartate
6.2.1.9
3.1.2.30
5.4.99.2
5.1.99.1
cis-Aconitate
2.3.3.9
(R)-Methyl-malonyl-CoA
(2R)-Ethyl-malonyl-CoA
2.8.3.26
Formate
3.1.2.10
Formyl-CoA
1.4.1.-
(S)-Malyl-CoA
5.4.99.63
Nitrogen metabolism
Imino-aspartate
Acetyl-CoA
5.1.99.1
1.2.3.4
2.8.3.16
1.3.1.7
6.2.1.1
(S)-Methyl-malonyl-CoA
(2S)-Methyl-succinyl-CoA
4.1.1.2
4.1.1.8
4.2.1.3
Ascorbate and aldarate metabolism
4.2.1.-
4.1.3.24
Acetate
1.3.8.12
L-erythro-3-Methylmalyl-CoA
2.8.3.2
Oxalyl-CoA
(2R,3S)-β-Hydroxy-aspartate
6.4.1.3
4.2.1.148
Mesaconyl-CoA
Oxalate
6.2.1.8
Propionyl-CoA
4.1.3.24
1.2.3.5
1.2.1.17
4.1.3.41
Glyoxylate
4.1.1.54
4.1.1.47
4.1.3.1
Isocitrate
Dihydroxy-fumarate
Tartronate semialdehyde
Succinate
Glycolate
1.1.99.14
1.1.1.92
1.1.1.60
Vitamin B6 metabolism
1.1.3.15
5.3.1.22
O2
H2O2
4.1.3.16
4-Hydroxy-2-oxoglutarate
1.2.1.21
4.1.3.42
Pyruvate
1.11.1.6
Purine metabolism
Hydroxy-pyruvate
Glycol-aldehyde
1.1.1.26
D-Glycerate
1.1.1.26
1.1.1.79
4.1.1.40
4.1.3.13
3-Oxalomalate
1.1.1.29
1.1.1.81
1.1.1.79
1.1.1.29
Oxaloacetate
3.1.3.18
2.3.3.7
3-Ethylmalate
2.7.1.165
2.7.1.31
1.1.1.77
1.1.1.-
Butanoyl-CoA
Phosphoglycolate
3-Phospho-D-glycerate
2-Phospho-D-glycerate
Ethane-1,2-diol
D-Ribulose 1,5-P2
4.1.1.39
Carbon fixation
2.3.3.12
3-Propylmalate
Pentanoyl-CoA
Carbon fixation
Dihydroxy-acetone-P
4.1.2.-
Ribulose-1P
3.1.3.109
Pentose phosphate pathway
2.2.1.5
2-Hydroxy-3-oxoadipate
2-Oxoglutarate
Glycolysis / Gluconeogenesis
L-Glutamine
Alanine, aspartate and glutamate metabolism
6.3.1.2
2-Oxoglutarate
1.4.7.1
L-Glutamate
Glycine
NH3
1.4.1.27
2.6.1.4
2.6.1.44
2.1.2.1
2.6.1.45
L-Serine
Cysteine and methionine metabolism
Glycine, serine and threonine metabolism
00630 4/9/25
(c) Kanehisa Laboratories

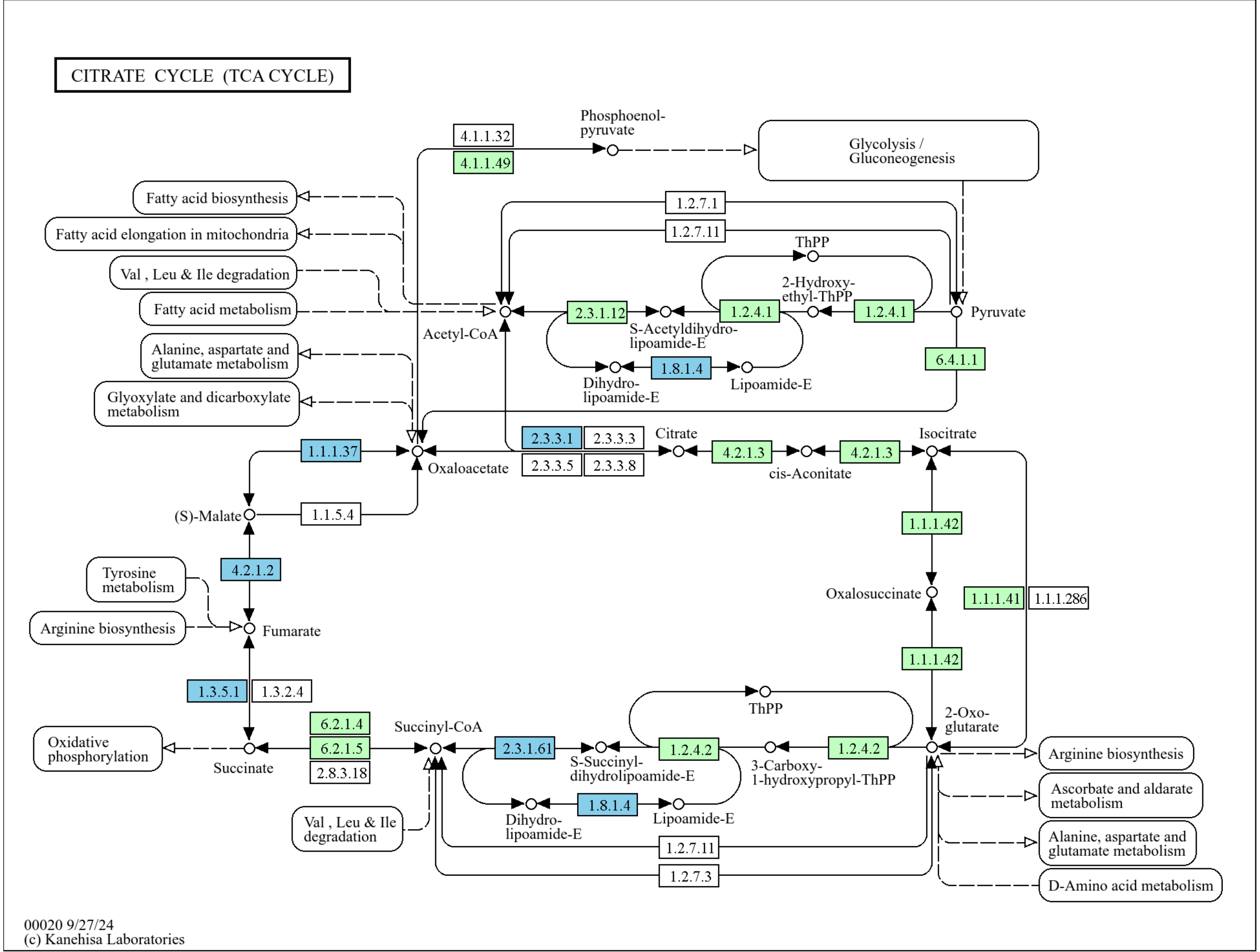
CITRATE CYCLE (TCA CYCLE)
4.1.1.32
4.1.1.49
Phosphoenol-
pyruvate
Glycolysis /
Gluconeogenesis
Fatty acid biosynthesis
Fatty acid elongation in mitochondria
Val , Leu & Ile degradation
Fatty acid metabolism
1.2.7.1
1.2.7.11
ThPP
2-Hydroxy-
ethyl-ThPP
Acetyl-CoA
2.3.1.12
1.2.4.1
1.2.4.1
Pyruvate
S-Acetyldihydro-
lipoamide-E
Alanine, aspartate and
glutamate metabolism
1.8.1.4
6.4.1.1
Dihydro-
lipoamide-E
Lipoamide-E
Glyoxylate and dicarboxylate
metabolism
2.3.3.1
2.3.3.3
2.3.3.5
2.3.3.8
Citrate
4.2.1.3
4.2.1.3
Isocitrate
cis-Aconitate
1.1.1.37
Oxaloacetate
(S)-Malate
1.1.5.4
1.1.1.42
4.2.1.2
Tyrosine
metabolism
Oxalosuccinate
1.1.1.41
1.1.1.286
Arginine biosynthesis
Fumarate
1.1.1.42
1.3.5.1
1.3.2.4
ThPP
6.2.1.4
Succinyl-CoA
2-Oxo-
glutarate
Oxidative
phosphorylation
6.2.1.5
2.3.1.61
1.2.4.2
1.2.4.2
Arginine biosynthesis
Succinate
2.8.3.18
S-Succinyl-
dihydrolipoamide-E
3-Carboxy-
1-hydroxypropyl-ThPP
Ascorbate and aldarate
metabolism
1.8.1.4
Dihydro-
lipoamide-E
Lipoamide-E
Val , Leu & Ile
degradation
Alanine, aspartate and
glutamate metabolism
1.2.7.11
1.2.7.3
D-Amino acid metabolism
00020 9/27/24
(c) Kanehisa Laboratories

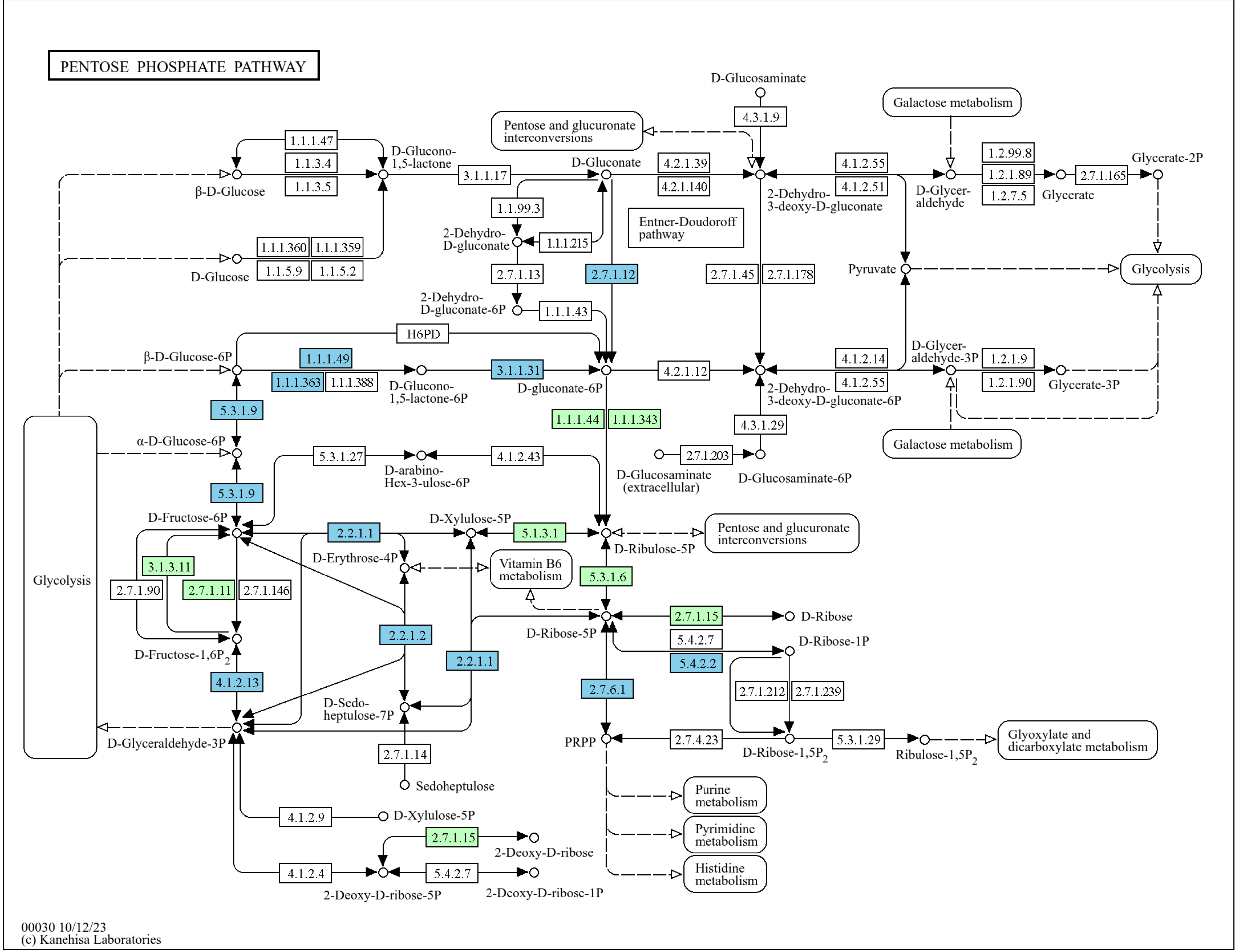
PENTOSE PHOSPHATE PATHWAY
D-Glucosaminate
4.3.1.9
Galactose metabolism
Pentose and glucuronate interconversions
1.1.1.47
1.1.3.4
1.1.3.5
D-Glucono-1,5-lactone
3.1.1.17
D-Gluconate
4.2.1.39
4.2.1.140
4.1.2.55
4.1.2.51
1.2.99.8
1.2.1.89
1.2.7.5
2.7.1.165
Glycerate-2P
β-D-Glucose
2-Dehydro-3-deoxy-D-gluconate
D-Glycer-aldehyde
Glycerate
1.1.99.3
Entner-Doudoroff pathway
2-Dehydro-D-gluconate
1.1.1.215
1.1.1.360
1.1.1.359
1.1.5.9
1.1.5.2
D-Glucose
2.7.1.13
2.7.1.12
2.7.1.45
2.7.1.178
Pyruvate
Glycolysis
2-Dehydro-D-gluconate-6P
1.1.1.43
H6PD
β-D-Glucose-6P
1.1.1.49
1.1.1.363
1.1.1.388
D-Glucono-1,5-lactone-6P
3.1.1.31
D-gluconate-6P
4.2.1.12
4.1.2.14
4.1.2.55
2-Dehydro-3-deoxy-D-gluconate-6P
D-Glycer-aldehyde-3P
1.2.1.9
1.2.1.90
Glycerate-3P
5.3.1.9
1.1.1.44
1.1.1.343
4.3.1.29
α-D-Glucose-6P
5.3.1.27
D-arabino-Hex-3-ulose-6P
4.1.2.43
2.7.1.203
D-Glucosaminate (extracellular)
D-Glucosaminate-6P
Galactose metabolism
5.3.1.9
D-Fructose-6P
2.2.1.1
D-Xylulose-5P
5.1.3.1
D-Ribulose-5P
Pentose and glucuronate interconversions
D-Erythrose-4P
Vitamin B6 metabolism
3.1.3.11
2.7.1.90
2.7.1.11
2.7.1.146
Glycolysis
5.3.1.6
2.7.1.15
D-Ribose
D-Ribose-5P
5.4.2.7
D-Ribose-1P
5.4.2.2
2.2.1.2
D-Fructose-1,6P2
2.2.1.1
4.1.2.13
2.7.6.1
2.7.1.212
2.7.1.239
D-Sedo-heptulose-7P
D-Glyceraldehyde-3P
2.7.1.14
PRPP
2.7.4.23
D-Ribose-1,5P2
5.3.1.29
Ribulose-1,5P2
Glyoxylate and dicarboxylate metabolism
Sedoheptulose
Purine metabolism
4.1.2.9
D-Xylulose-5P
Pyrimidine metabolism
2.7.1.15
2-Deoxy-D-ribose
Histidine metabolism
4.1.2.4
5.4.2.7
2-Deoxy-D-ribose-5P
2-Deoxy-D-ribose-1P
00030 10/12/23
(c) Kanehisa Laboratories

Supplementary Figure 4. Predicted metabolic pathways associated with energy metabolism and redox balance in *Debaryomyces hansenii* under YNBG + BaP treatment. Enzymes absent in the genome are shown in white, enzymes present but not expressed are indicated in green, and enzymes overexpressed in BaP-treated cultures are shown in blue.
