# Supplementary material for: Transcriptomic profiling of Debaryomyces hansenii reveals detoxification and stress responses to benzo(a)pyrene exposure
Source: Appl Environ Microbiol. 2025 Sep 16;91(10):e01557-25. doi: 10.1128/aem.01557-25 (PMC12542653; doi:10.1128/aem.01557-25)
Supplement: Figure S5 — Metabolites proposed by GC-MS analysis as potential products of BaP catabolism by Debaryomyces hansenii. [file aem.01557-25-s0005.pdf]

Supplementary Figure 5. Metabolites proposed by GC-MS as a result of BaP catabolism by *D. hansenii*.

n-hexadecanoic acid

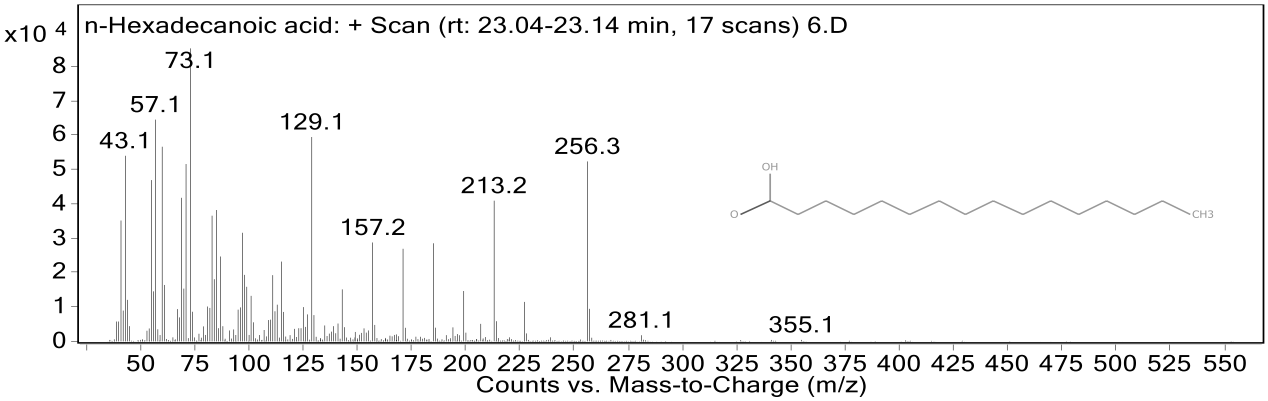

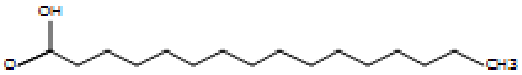

Phenol, 2,2'-methylenebis[6-(1,1-dimethylethyl)-4-ethyl

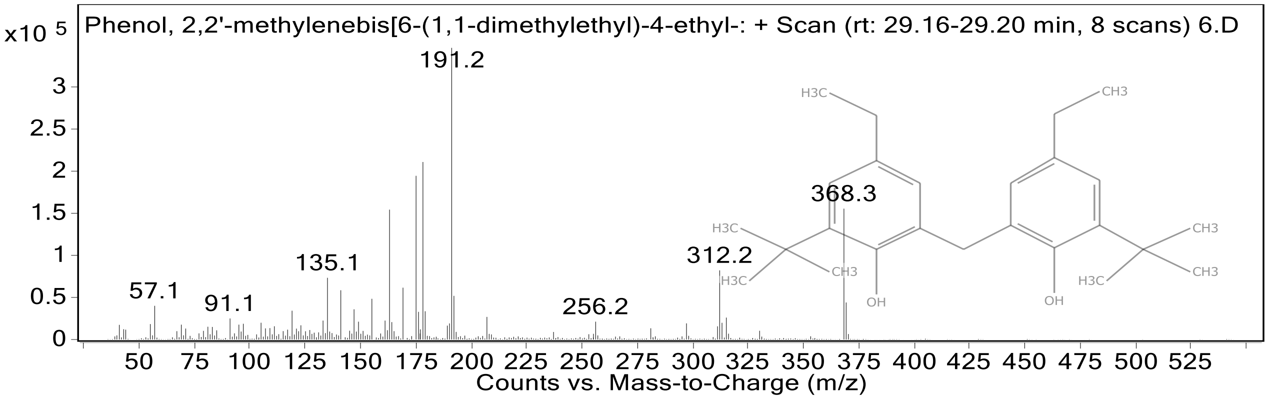

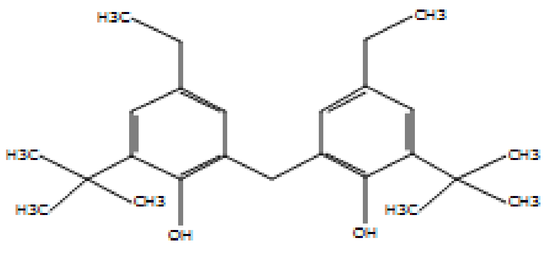

Phthalic acid, di(2-propylpentyl) ester

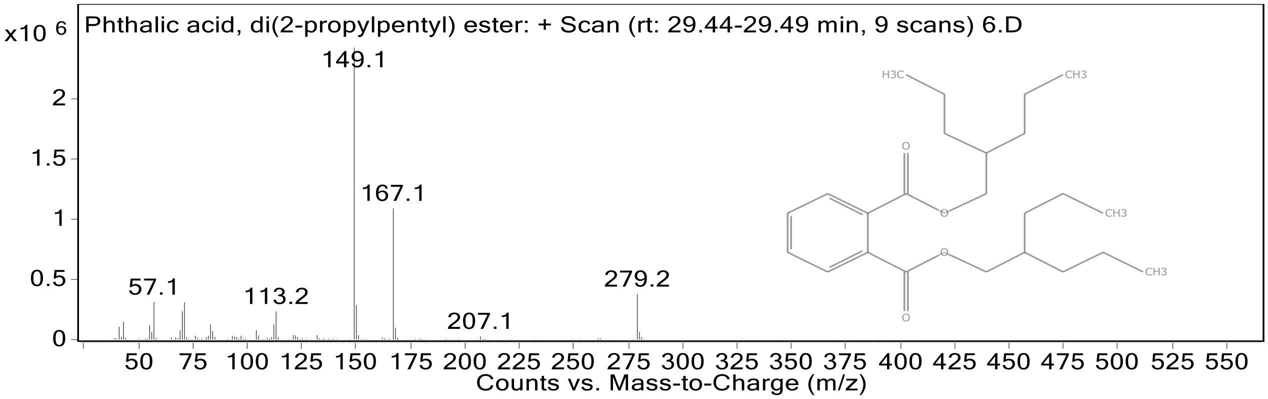

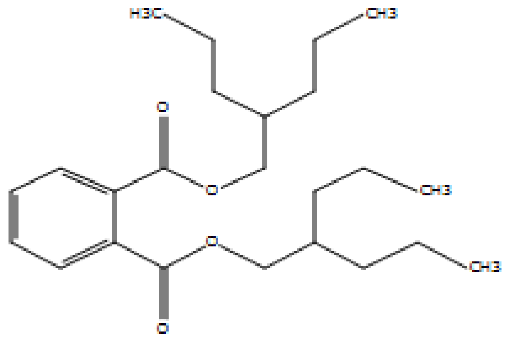

1,3-Benzenedicarboxylic acid, bis(2-ethylhexyl) ester

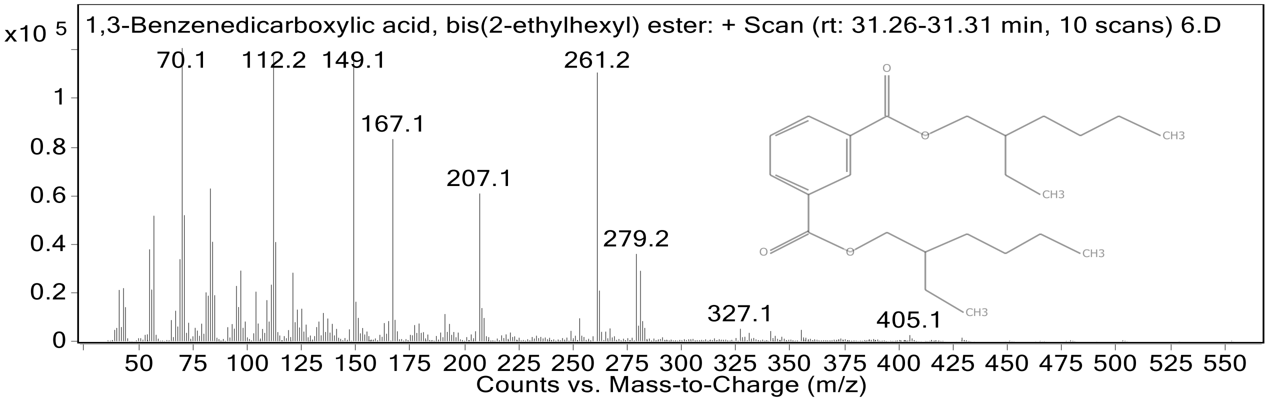

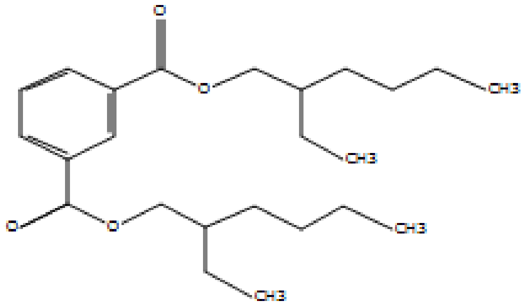

Bis(2-ethylhexyl) phthalate

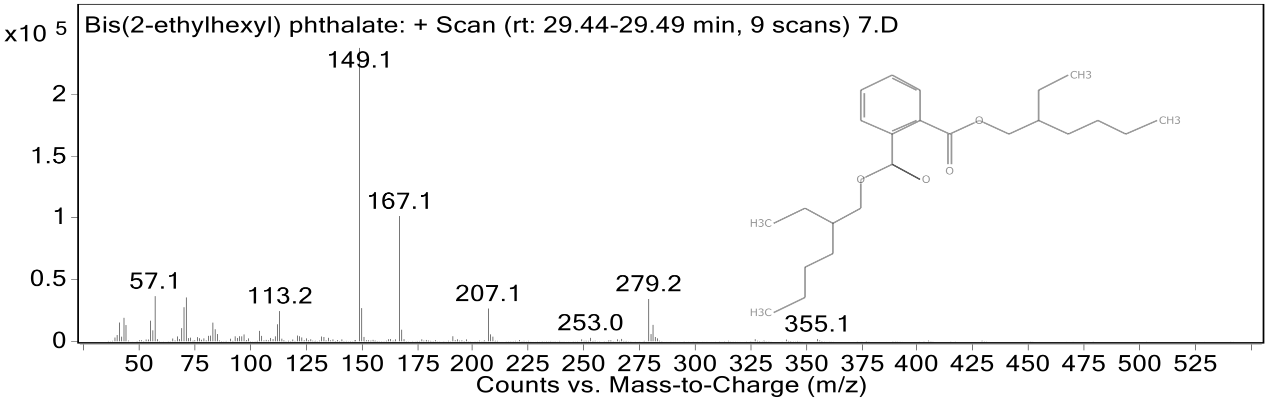

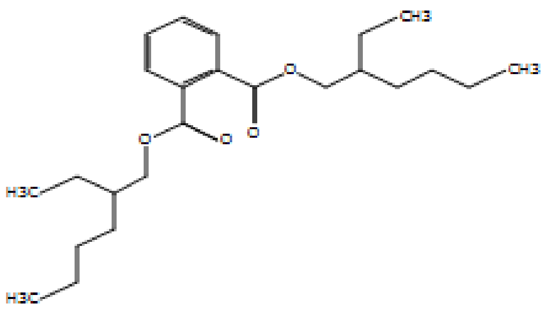

Phthalic acid, butyl tetradecyl ester

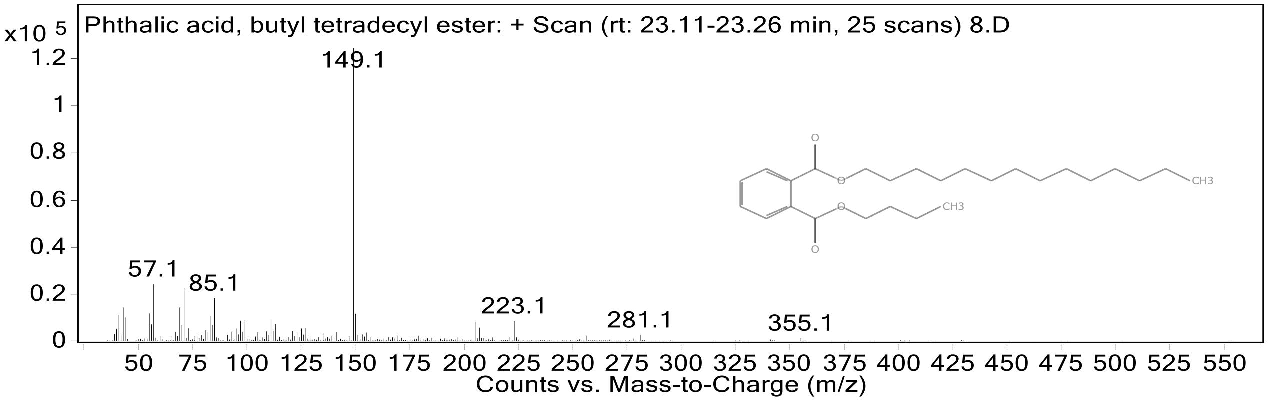

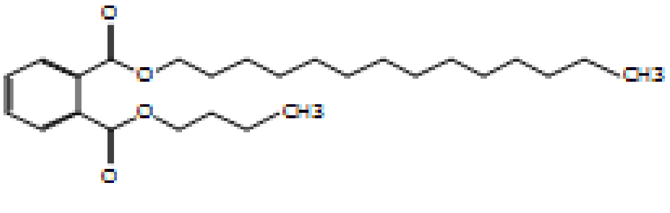

Phenol, 2,6-bis(1,1-dimethylethyl)

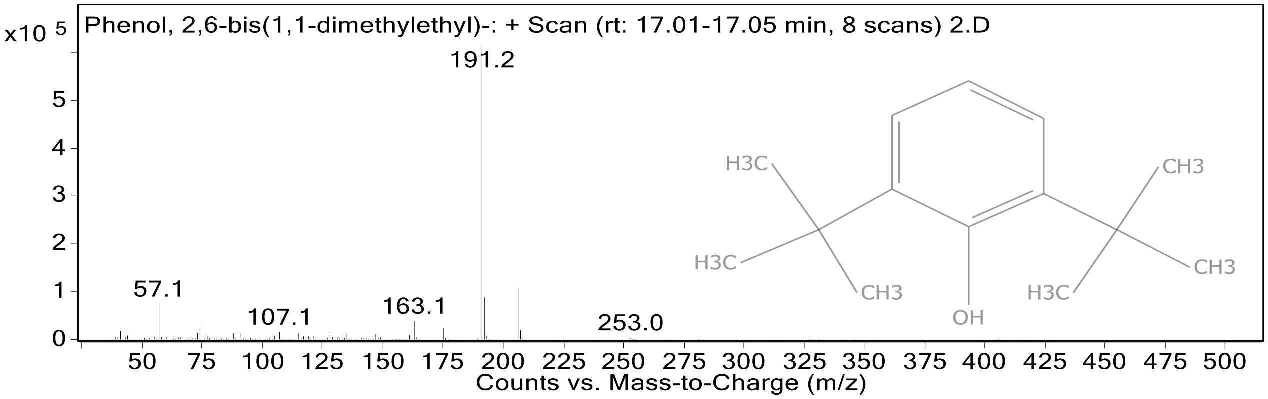

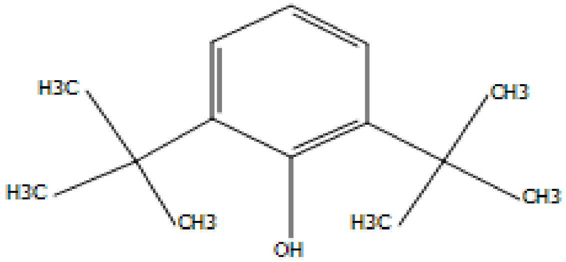

Phenol, 2,5-bis(1,1-dimethylethyl)

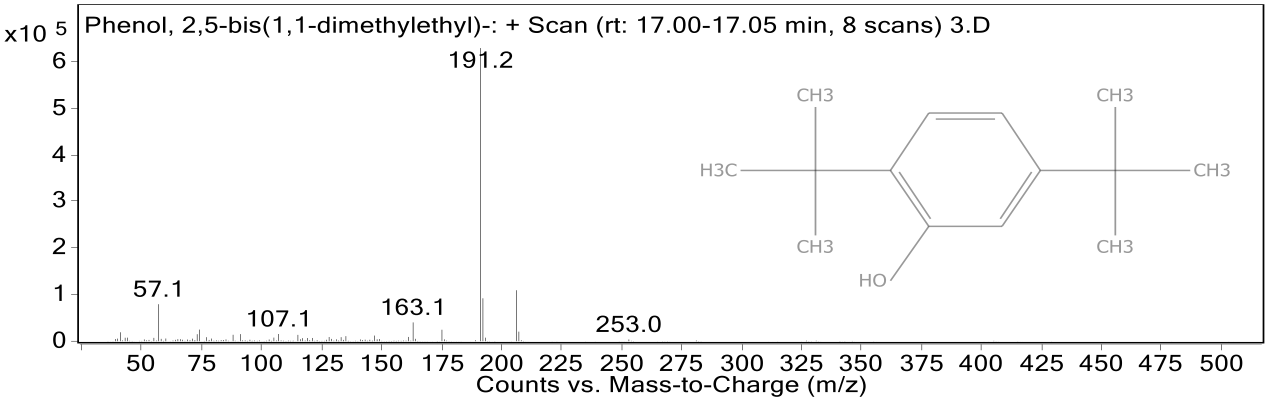

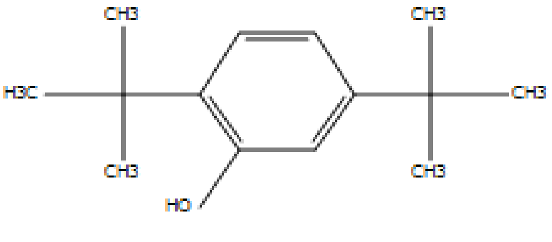

Benzyl Benzoate

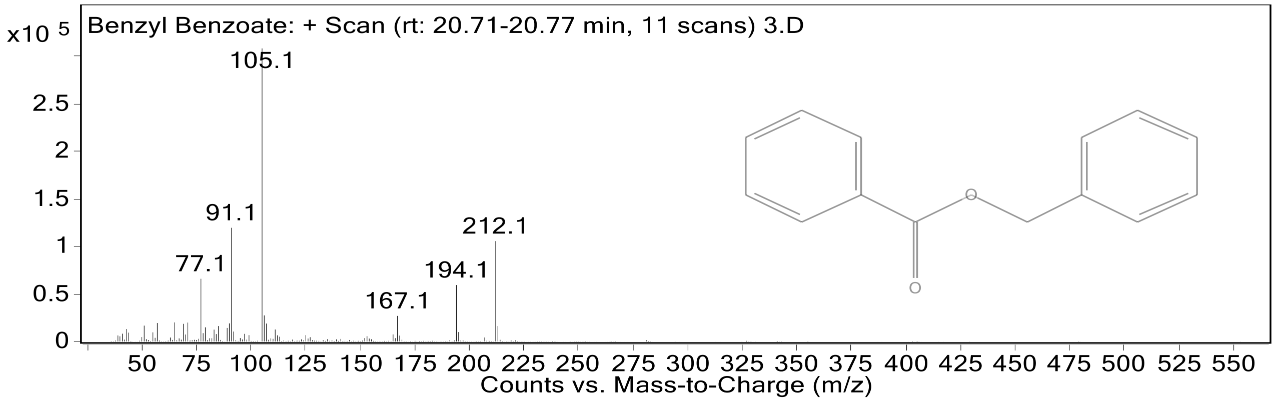

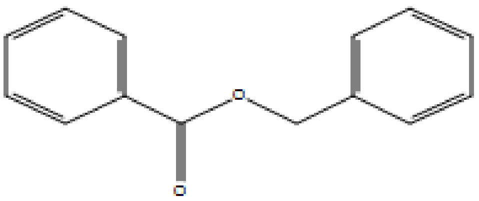

1,2-Benzenedicarboxylic acid, butyl 8-methylnonyl ester

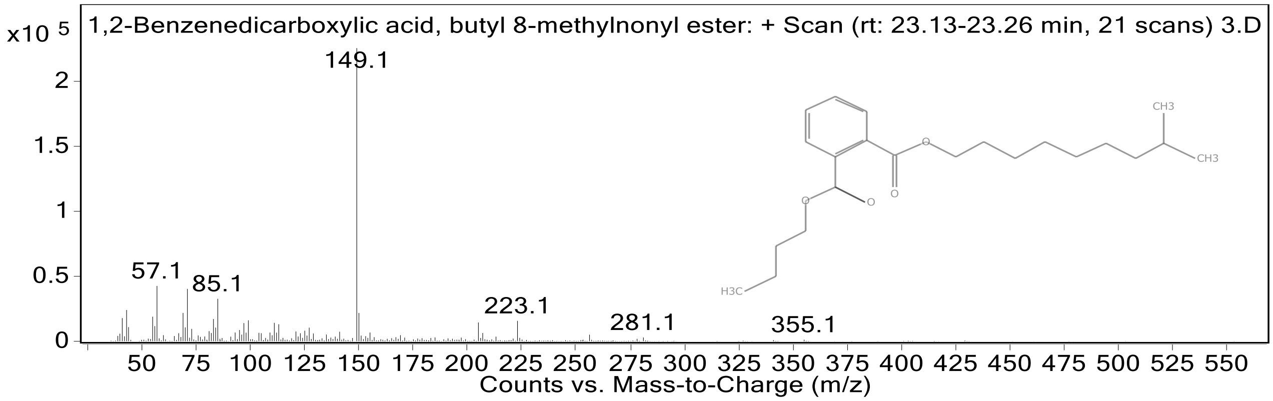

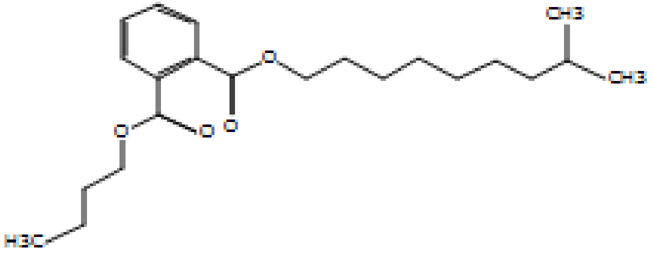

Octadecanoic acid

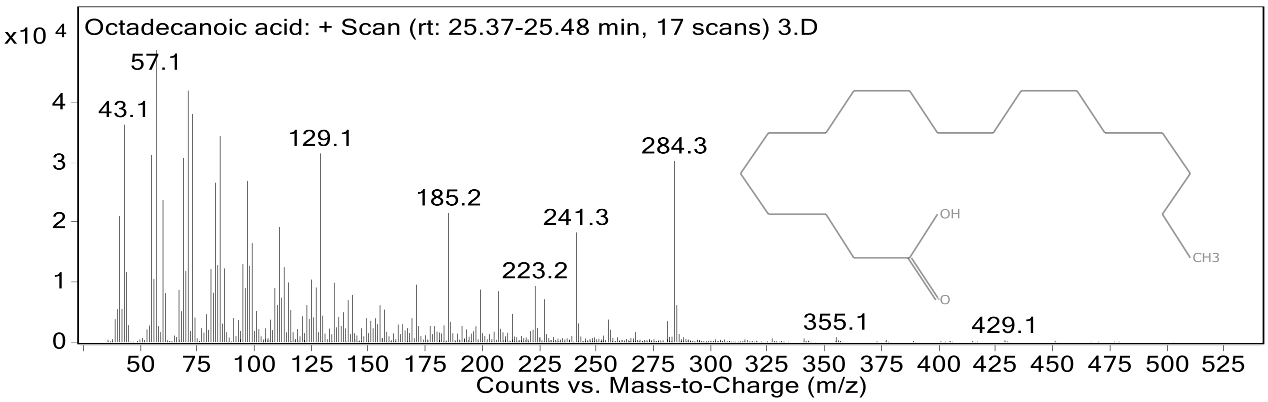

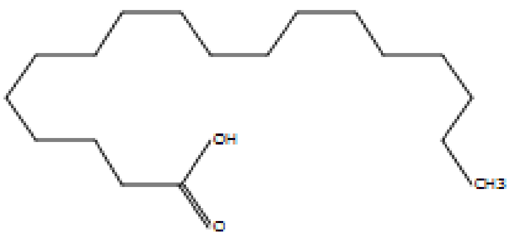

2,4-Di-tert-butylphenol

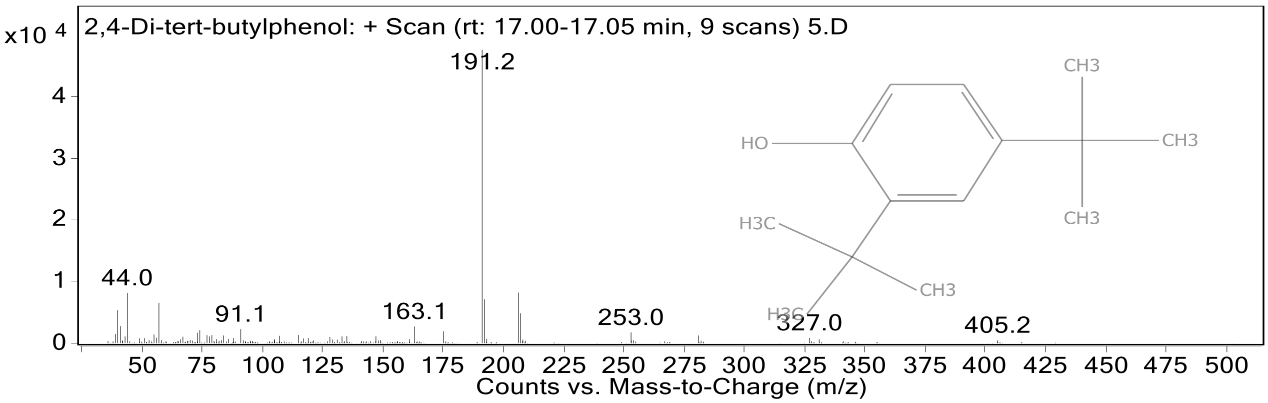

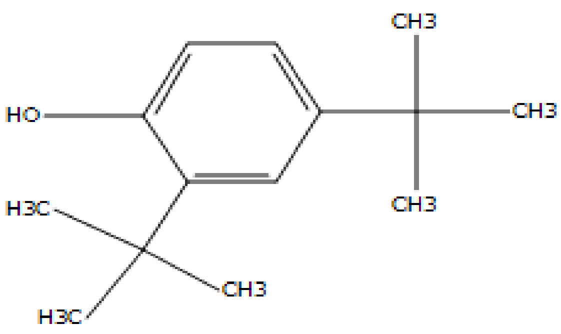

Supplementary Figure 5. Metabolites proposed by GC–MS analysis as potential products of BaP catabolism by *Debaryomyces hansenii*. Identified compounds include n-hexadecanoic acid, phenol derivatives [e.g., 2,2′-methylenebis(6-tert-butyl-4-ethyl)phenol; 2,6-di-tert-butylphenol; 2,5-di-tert-butylphenol], phthalic acid esters [e.g., di(2-propylpentyl) phthalate; bis(2-ethylhexyl) phthalate; butyl tetradecyl phthalate; bis(2-ethylhexyl) 1,3-benzenedicarboxylate; butyl 8-methylnonyl 1,2-benzenedicarboxylate], benzyl benzoate, octadecanoic acid, and 2,4-di-tert-butylphenol.
